# Beyond the Steroid Trial: A Scoping Review of Biomarkers for Pediatric Nephrotic Syndrome

**DOI:** 10.3390/medsci14040448

**Published:** 2026-07-29

**Authors:** Tudor-Ilie Lazaruc, Anca-Lavinia Lazaruc-Postolache, Iuliana-Magdalena Starcea, Roxana-Alexandra Bogos, Maria-Adriana Mocanu, Madalina-Andreea Beldie, Ingrith-Crenguta Miron

**Affiliations:** 1 Pediatrics Department, University of Medicine and Pharmacy Grigore T. Popa Iasi, 700115 Iasi, Romania; tudor.lazaruc@umfiasi.ro (T.-I.L.); anca.cianga@umfiasi.ro (A.-L.L.-P.); maria-adriana.mocanu@umfiasi.ro (M.-A.M.); madalina-andreea.beldie@umfiasi.ro (M.-A.B.); ingrith.miron@umfiasi.ro (I.-C.M.); 2 Emergency Clinical Hospital for Children “St. Mary” Iasi, 700309 Iasi, Romania

**Keywords:** nephrotic syndrome, pediatric, biomarkers, anti-nephrin autoantibodies, steroid resistance, podocytopathy, scoping review

## Abstract

**Background:** Pediatric idiopathic nephrotic syndrome (INS) is classified primarily by corticosteroid response, delaying identification of steroid-resistant disease and exposing children to unnecessary treatment toxicity. Novel biomarkers could enable earlier biological stratification and treatment guidance. **Objectives:** The study aimed to map available evidence on candidate biomarkers in pediatric INS published since 2020, with emphasis on anti-nephrin autoantibodies and their potential for clinical translation. **Data Sources:** PubMed/MEDLINE and Web of Science Core Collection (January 2020–October 2025), with a supplementary verification search in Scopus and Embase and manual reference screening. **Eligibility Criteria:** Original studies reporting circulating, urinary, or tissue-based biomarkers in children aged 0–18 years with idiopathic NS, with outcomes related to diagnosis, treatment response, relapse prediction, or monitoring. Studies in adults only, secondary NS, or animal models were excluded. **Results:** After screening, 34 studies met the eligibility criteria and were included, grouped into five categories: autoantibodies, urinary markers, immune cell signatures, cytokines/chemokines, and exploratory markers (metabolomics, extracellular vesicles, lipid profiles, microRNAs). Anti-nephrin IgG emerged as the most mechanistically informative marker, with seroprevalence declining across phenotypes (SSNS 68%, SDNS 28%, non-genetic SRNS 14%, genetic SRNS 2%) and positivity predicting response to intensified immunosuppression. Of the candidates reviewed, urinary NGAL and peripheral B-cell subset monitoring are the most readily implementable with existing laboratory infrastructure. **Conclusions:** Pediatric INS encompasses a spectrum of immune-mediated podocytopathies that may soon be distinguishable by emerging biomarker profiles. Anti-nephrin autoantibodies provide the strongest mechanistic evidence for an autoimmune podocytopathy.

## 1. Introduction

Childhood idiopathic nephrotic syndrome (INS) is the most common glomerular disease in pediatrics and remains a major driver of morbidity due to edema, infections, thromboembolic events, growth, and metabolic complications from corticosteroid exposure. Contemporary estimates place the mean global incidence near 3 per 100,000 children per year, with substantial geographic and ethnic variability and frequent relapses that shape long-term burden and care needs: a 2021 meta-analysis (27 incidence cohorts; 54 relapse cohorts) reported a pooled incidence of 2.92 per 100,000 children/year and an overall relapse risk of about 72% [1], underscoring both the rarity and the relapse-prone nature of the condition. Epidemiologic data also demonstrate marked ethnic variation, with South Asian children experiencing a more than sixfold higher incidence of nephrotic syndrome compared to European peers [2]. Interestingly, despite this higher incidence, South Asian and East/Southeast Asian children showed fewer relapses than European children, suggesting potential genetic or environmental modifiers of disease course [2]. Recent educational reviews echo these figures and emphasize that advances in disease biology (e.g., immunologic and podocyte pathways) are beginning to inform practice and guideline evolution [3].

First-line therapy for new-onset INS is corticosteroids, with most children achieving initial remission; however, a substantial proportion will subsequently relapse, and an important minority develop steroid dependence or resistance. Relapse-driven exposure to cumulative steroids and additional immunosuppressants introduces toxicity risks, fueling the need for tools that better stratify risk, forecast relapse, and individualize treatment. In this context, evidence-based guidance is essential to harmonize terminology and treatment strategies internationally.

Three major international guidelines currently shape pediatric nephrotic syndrome care: the IPNA 2020 recommendations for SRNS, the IPNA 2022 recommendations for SSNS, and the KDIGO 2025 guideline that integrates both disease categories [4,5,6]. Similarities and nuances among these guidelines are summarized in Table 1.

Both IPNA and KDIGO underscore the limitations of current classification, which is based largely on treatment response, and the potential of biomarkers to refine definitions and guide therapy.

### 1.1. Pathophysiology

A clear understanding of the cellular and molecular mechanisms driving proteinuria provides the rationale for why certain biomarkers are of interest, how they might reflect disease activity, and in which clinical contexts they could offer the greatest utility.

**Figure 1:**
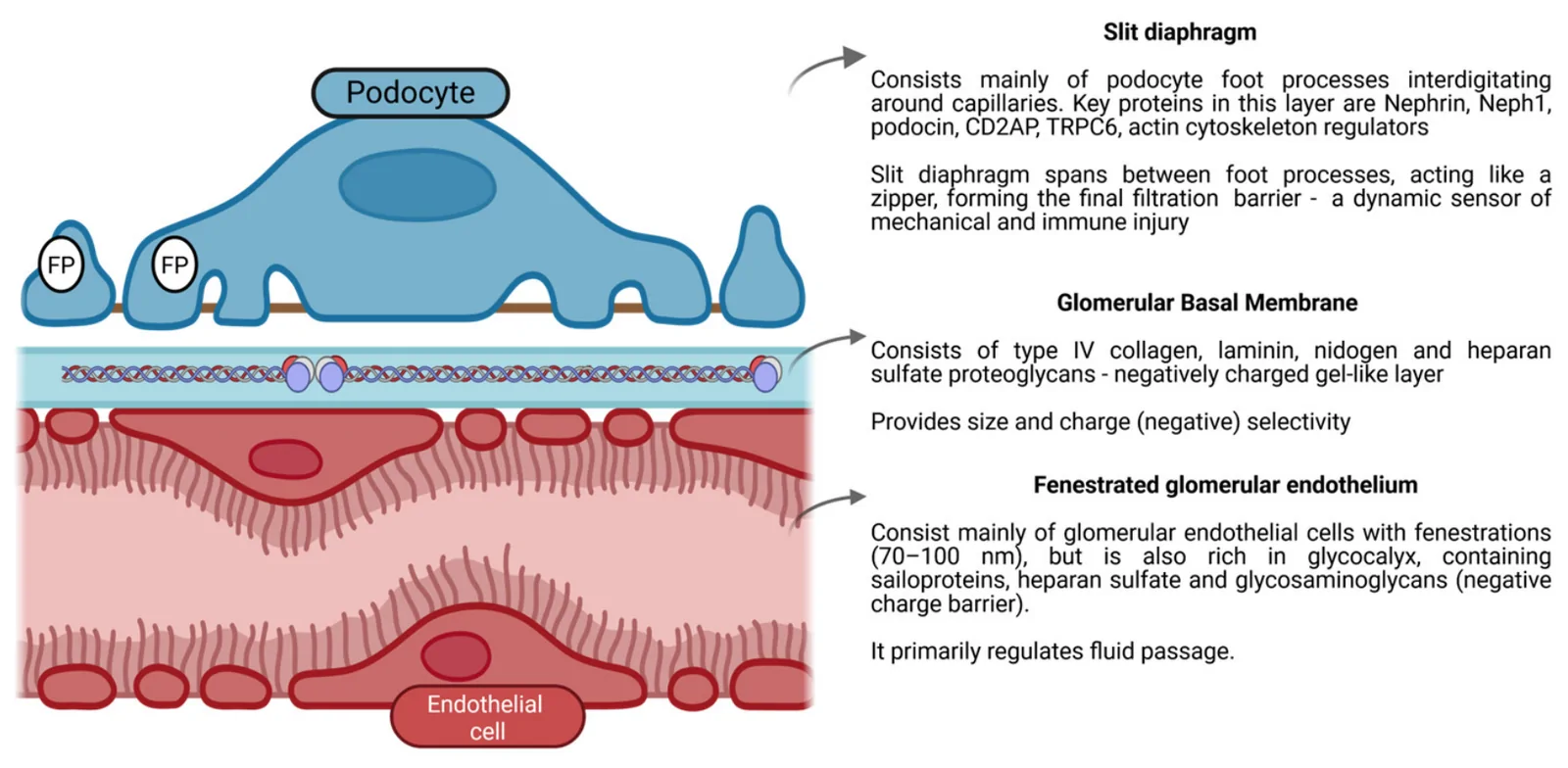
Glomerular filtration barrier consisting of the fenestrated glomerular endothelium, the glomerular basement membrane, and the podocyte foot processes (FP) interconnected by the slit diaphragm.

The glomerular filtration barrier (Figure 1) is composed of three interdependent layers: the fenestrated endothelium with its glycocalyx that restricts cells and contributes to charge selectivity; the glomerular basement membrane (GBM), a dense network of type IV collagen, laminin, and heparan sulfate proteoglycans providing structural support and size/charge filtration, and the podocyte foot processes interconnected by the slit diaphragm-a protein complex (including nephrin, podocin, and CD2AP) that serves as the final barrier to albumin leakage. Together, these layers form a highly specialized sieve whose disruption leads to proteinuria in nephrotic syndrome.

In children, most idiopathic cases behave like primary podocytopathy: immune signals (rather than fixed scarring) disrupt podocyte shape, detach foot processes, and loosen the slit diaphragm, so albumin slips through. Mechanistically, two aspects dominate: (1) immune/circulating factors that transiently alter podocyte biology, and (2) intrinsic podocyte pathway changes (cytoskeleton, slit diaphragm signaling).

Recent work ties these two together. On one hand, Hengel et al. [7] identified anti-nephrin autoantibodies in about 44% of adults with minimal change disease (*n* = 105) and 9% with primary FSGS (*n* = 74), with titers tracking disease activity and kidney tissue showing antibody binding at the slit diaphragm—a direct immune trigger of podocyte dysfunction and a biomarker candidate [7]. On the other hand, Vivarelli et al.’s [8] review on childhood nephrotic syndrome emphasizes how T- and B-cell dysregulation and circulating permeability factors converge on podocyte injury, yet vary among patients, explaining why some are steroid sensitive while others relapse frequently or develop resistance [8].

In a different approach, Purohit et al. [9] outlined how candidate mediators (cytokines, immune factors) perturb podocyte cytoskeletal and signaling pathways, producing the ultrastructural lesion of foot-process effacement, the morphological hallmark of minimal change disease [9]. Beyond immune-podocyte crosstalk, other mechanisms matter: Frățilă et al. [10] described how both the underfill hypothesis (low oncotic pressure → RAAS activation and sodium retention) and the overfill hypothesis (primary tubular sodium retention) explain edema, showing why diuretic strategies must be individualized.

Bringing this together, Vaz de Castro et al. [11] frame nephrotic syndrome as a podocytopathy, where diverse genetic, immune, and environmental insults converge on podocyte injury and slit-diaphragm disruption. This unifying view helps clarify where biomarkers should be sought: (i) immune–podocyte interface markers (e.g., anti-nephrin antibodies) to capture immune activity, (ii) podocyte injury markers to reflect stress downstream, and (iii) systemic mediators of immune activation and sodium retention. Known mechanisms already explain why steroids work in many children—what remains under study is which signals dominate in which patients, and how to map them to precise biomarkers.

### 1.2. Scope of the Review

This scoping review aims to synthesize current evidence on novel and emerging biomarkers in pediatric idiopathic nephrotic syndrome, with particular emphasis on their potential clinical utility in diagnosis, prognosis, and treatment guidance. While a wide range of candidate biomarkers has been proposed, the focus of this article is to critically appraise their relevance in children, where validation remains limited compared to adults. Special attention is given to anti-nephrin autoantibodies, a recently described immune biomarker that directly links podocyte injury to disease activity and relapses in subsets of patients.

## 2. Materials and Methods

This scoping review was conducted in accordance with the methodological framework for scoping reviews and is reported following the PRISMA Extension for Scoping Reviews (PRISMA-ScR) [12]. The review was not registered. A systematic literature search was conducted in PubMed/MEDLINE and Web of Science Core Collection. To address the completeness of the primary search, a supplementary verification search using semantically equivalent search strings, the identical time window (January 2020–October 2025), and the identical eligibility criteria were subsequently performed in Scopus and Embase. In addition, the reference lists of included articles and of relevant reviews were screened manually to capture further eligible studies.

The search strategy combined free-text keywords and controlled vocabulary (MeSH terms in PubMed) related to the population (pediatric nephrotic syndrome), the exposure of interest (biomarkers), and specific targets (anti-nephrin antibodies, suPAR, CD80, urinary markers, circulating factors). Boolean operators (AND, OR, NOT) were applied to refine results, and database-specific filters were used to restrict the search to human studies, English language, and children (0–18 years) published from January 2020 onwards. For PubMED, the search string was “nephrotic syndrome” AND (child* OR pediatric) AND (biomarker OR anti-nephrin OR antibody OR autoantibody OR proteomics OR suPAR OR CD80 OR “urinary marker” OR urinary OR “circulating factor”) NOT lupus NOT nephritis NOT membranous. For Web of Science, the search string was (“nephrotic syndrome”) AND (child* OR pediatric* OR paediatric* OR adolescent*) AND (biomarker* OR “anti-nephrin” OR nephrin OR antibody* OR autoantibody* OR proteomic* OR suPAR OR CD80 OR “urinary marker*” OR “circulating factor*”) NOT (lupus OR nephritis OR membranous). 

The Scoping Reviews (PRISMA-ScR) Checklist are shown in Table S6 (Supplementary Data).

### 2.1. Screening, Reviewer Calibration, and Data Extraction

Records were exported to a shared reference manager and de-duplicated. Prior to formal screening, the two screening reviewers (T.I.L, A.L.L.P) performed a calibration exercise on a random sample of records, independently applying the eligibility criteria and comparing decisions; discrepancies were discussed and the operational definitions refined before screening of the full set began. Titles and abstracts were then screened independently and in duplicate, and all records judged potentially eligible by either reviewer proceeded to independent full-text assessment. Data were extracted using a standardized form developed a priori and piloted before application, capturing first author and year; country and setting; study design; participant number, age range, and nephrotic syndrome phenotype; control or comparator group; disease state and immunosuppressive exposure at sampling; the biomarker and biological matrix; the analytical platform; the reported clinical outcome; and all quantitative performance metrics. Extraction was performed by one reviewer and independently verified against the source article by a second. Disagreements at any stage were resolved by discussion, with a senior reviewer (I.M.S) arbitrating unresolved cases. No automation or machine-learning screening tools were used. Consistent with the scoping design and the heterogeneity of biomarkers, assays, and outcome definitions, findings were synthesized narratively and no meta-analysis was attempted.

### 2.2. Appraisal of the Evidence

Formal critical appraisal of individual sources is not a mandatory component of a scoping review, whose purpose is to map the extent and nature of an evidence base rather than to grade it. Nevertheless, because the candidate biomarkers reviewed here differ substantially in the robustness of their supporting evidence, each included study was appraised against six pre-specified domains: study design (prospective/longitudinal versus retrospective/cross-sectional); sample size, with fewer than 50 pediatric participants considered a substantive constraint; single- versus multicenter recruitment; the presence of an appropriate control or comparator group; whether the biomarker assay was described in reproducible detail; and whether the reported associations were internally or externally validated. On this basis each study was assigned to one of three tiers of evidential strength. Tier A denotes multicenter and/or prospective studies that are adequately powered, include an appropriate comparator, and report validated or longitudinally corroborated findings. Tier B denotes methodologically sound single-center studies with adequate sample size and appropriate controls but without independent validation. Tier C denotes exploratory or discovery-phase studies, typically with fewer than 50 participants or unvalidated high-dimensional analyses, whose findings are hypothesis-generating. Appraisal was performed independently by two reviewers (T.I.L, A.L.L.P) with disagreements resolved by consensus, and the tier assigned to each study is reported in Supplementary Tables S1–S5. This appraisal is offered as a transparent guide to evidential strength rather than as a formal risk-of-bias instrument; no study was excluded based on its appraisal.

### 2.3. Inclusion Criteria

Studies were considered eligible for inclusion if they met the following criteria: (1) population: children and adolescents (0–18 years) diagnosed with INS, including steroid-sensitive (SSNS), steroid-resistant (SRNS), frequently relapsing (FRNS), or steroid-dependent (SDNS) forms; (2) study design: original research articles, including clinical trials, cohort studies, case-control studies, cross-sectional studies, and mechanistic translational studies; (3) exposure/biomarker: assessment of circulating (e.g., anti-nephrin autoantibodies, suPAR, cytokines), urinary (e.g., CD80, proteomic or metabolomic signatures), or podocyte/tissue-based molecular markers with relevance to clinical course; (4) outcomes: reporting an association of the biomarker(s) with diagnosis, treatment response, relapse prediction, prognosis, or disease monitoring; and (5) publication characteristics: published in peer-reviewed journals from January 2020 to October 2025, available in English.

### 2.4. Exclusion Criteria

**Figure 2:**
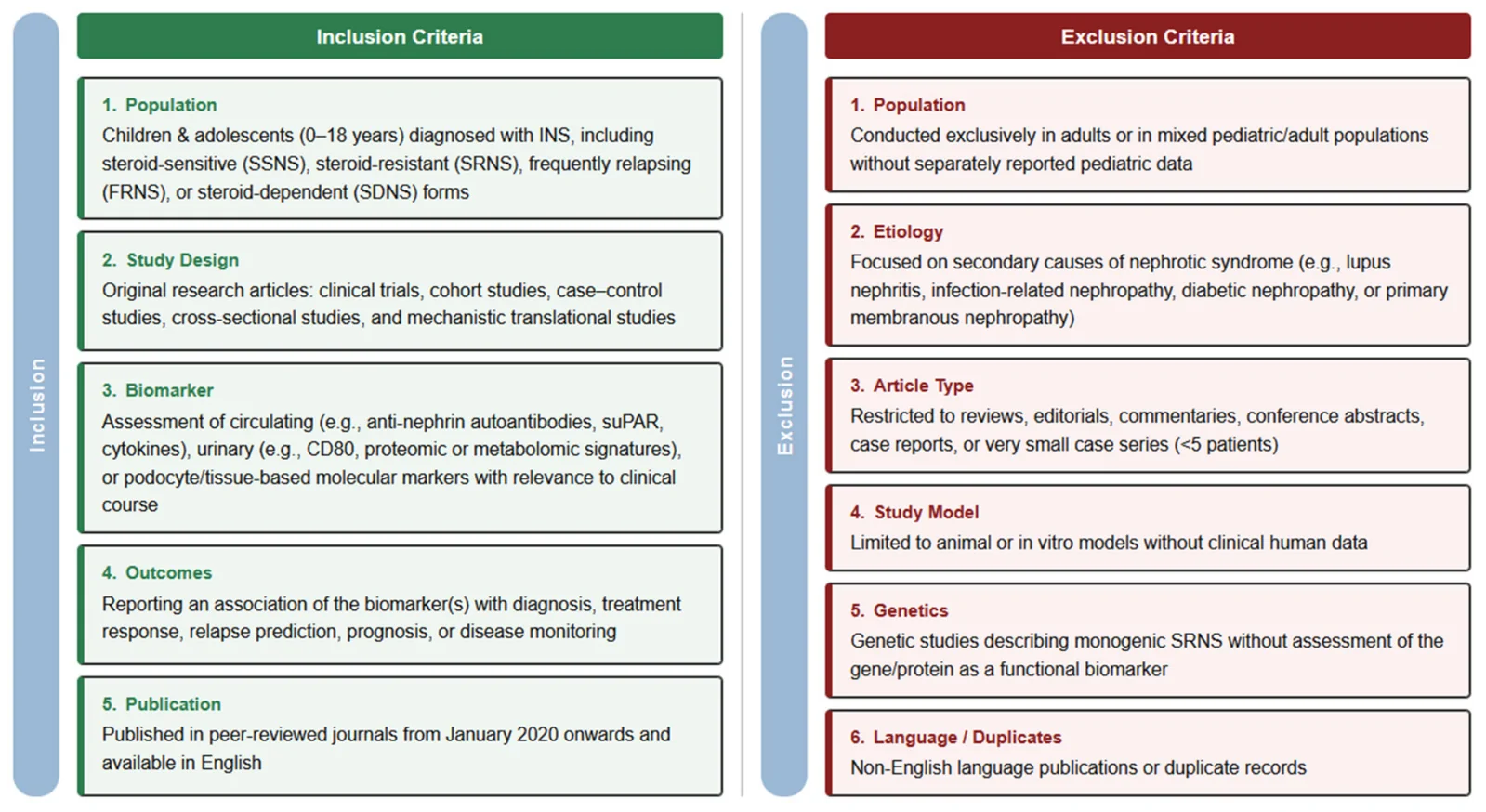
Inclusion and exclusion criteria.

Studies were excluded if they met any of the following criteria: (1) conducted exclusively in adults or in mixed pediatric/adult populations without separately reported pediatric data; (2) focused on secondary causes of nephrotic syndrome (e.g., lupus nephritis, infection-related nephropathy, diabetic nephropathy, or primary membranous nephropathy); (3) article type restricted to reviews, editorials, commentaries, conference abstracts, case reports, or very small case series (<5 patients); (4) studies limited to animal or in vitro models without clinical human data; (5) genetic studies describing monogenic SRNS without assessment of the gene/protein as a functional biomarker; and (6) non-English language or duplicate records. See Figure 2 for a general overview of inclusion and exclusion criteria.

## 3. Results

**Figure 3:**
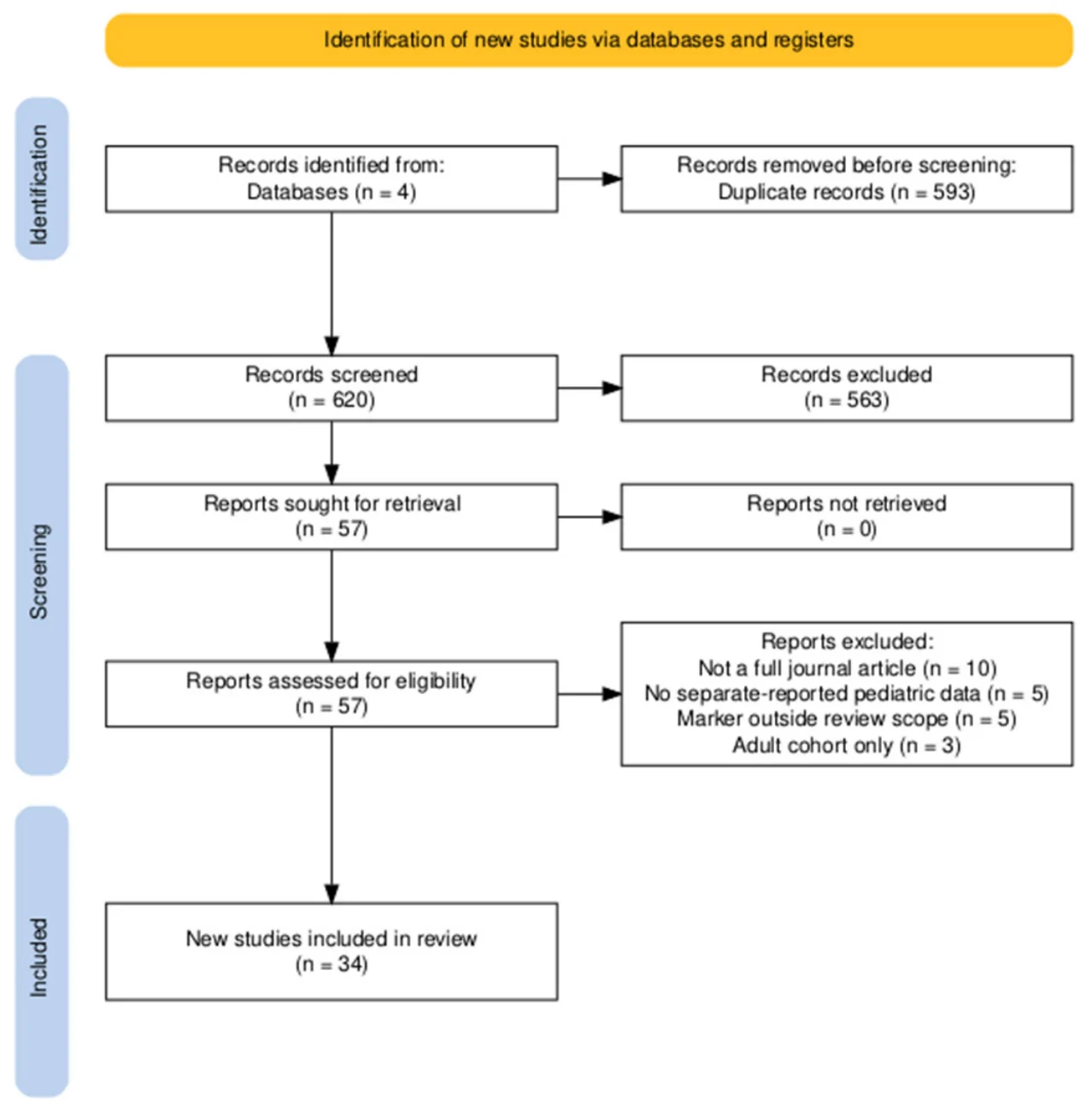
PRISMA flowchart [13].

The systematic search in PubMed, Web of Science, Embase, and Scopus yielded a total of 1213 records. After removal of duplicates and screening of titles and abstracts, 57 records underwent full-text assessment, and 34 studies met the eligibility criteria and were included in the present review (Figure 3).

To facilitate synthesis and comparison, the 34 included studies were grouped according to the type of biomarker investigated. Five main categories emerged from this process: autoantibodies, urinary markers, immune cell signatures, cytokines, and chemokines, and a broader group of other biomarkers encompassing metabolites, microRNAs, extracellular vesicles, and markers of oxidative stress. This framework provided a logical structure for examining the evidence and highlighting patterns across different biomarker domains; therefore, the Section 3 is organized accordingly. Table 2 summarizes the most clinically relevant markers; each is detailed by category below.

### 3.1. Autoantibodies

Six studies evaluated podocyte-related autoantibodies in pediatric nephrotic syndrome [7,14,15,16,17].

Ye et al. (2021) [17] screened sera from 341 children with idiopathic NS and identified seven novel podocyte autoantibodies (vinculin, SRSF9, proteasome subunit α1, aconitate hydratase 2, peptidyl-prolyl isomerase D, peroxiredoxin 1, and F-actin capping protein β). At least one antibody was detected in 66% of cases, with anti-vinculin being most frequent (56%). Autoantibody-positive patients had higher proteinuria, and titers declined after remission in steroid-sensitive cases but remained elevated in steroid-resistant disease.

Liu et al. (2025) [38] extended the autoantibody landscape by characterizing serum autoantibodies against proteasome subunit alpha type 1 (PSMA1) in 54 children with newly diagnosed INS, compared against normal controls and four disease-control groups (Kawasaki disease, Henoch–Schönlein purpura, HSP nephritis, and IgA nephropathy; *n* = 50 each). Anti-PSMA1 levels were significantly higher in INS than in every comparator group, correlated positively with proteinuria (r = 0.40), and declined in remission. A cut-off of 28.2 grayscale units discriminated INS with an AUC of 0.904 (sensitivity 74.1%, specificity 98.0%), reproduced in an independent validation cohort of 30 children. Punctate IgG co-localizing with PSMA1 was demonstrated in patient biopsies, and immunization, knockdown, and psma1-knockout models produced podocyte injury and proteinuria. Anti-PSMA1 thus represents a further member of the expanding podocyte-autoantibody family, reinforcing the concept of autoimmune podocytopathy while remaining, like the other podocyte autoantibodies, without a clinically available assay.

Watts et al. (2022) [14] examined 62 patients with minimal change disease and reported anti-nephrin IgG in 29% of patients during relapse, while absent in 30 healthy and 54 disease controls. In all 11 paired samples, antibodies present at relapse disappeared in remission. Immunoprecipitation confirmed nephrin binding only in relapse-phase sera, and biopsies from seropositive patients demonstrated punctate IgG deposits co-localized with nephrin.

Raglianti et al. (2024) [15] investigated 128 pediatric biopsies with minimal change or FSGS lesions. IgG deposition along the slit diaphragm was detected in 30% of minimal change and 25% of FSGS cases. Among positive cases, 77.8% showed nephrin co-localization, while the remainder suggested other slit diaphragm antigens. In steroid-resistant patients, positivity was observed in 27% of primary SRNS and 87.5% of secondary SRNS. Remission with second-line immunosuppression was achieved in 92.3% of antibody-positive versus 20% of antibody-negative cases. None of the antibody-positive patients progressed to kidney failure, compared to 51.7% of antibody-negative patients.

In two complementary multicenter studies, Hengel et al. [7,16] further characterized anti-nephrin autoantibodies across the full spectrum of pediatric NS. In the first [7], optimized for immunoprecipitation (IP) in a cohort of 182 children with INS, 94/182 (52%) were anti-nephrin positive overall, rising to 78/98 (80%) in nephrotic-range proteinuria and 35/39 (90%) when actively nephrotic without immunosuppression; only 1/50 pediatric controls was positive. Longitudinally, anti-nephrin levels moved with proteinuria, and the group developed a hybrid IP → ELISA for quantitation; no diagnostic sensitivity or specificity was formally reported. In the second study [16], conducted within the PodoNet consortium across all NS subtypes (*n* = 333), positivity was 69/101 (68%) in SSNS, 19/67 (28%) in SDNS, 14/103 (14%) in non-genetic SRNS, and 1/62 (2%) in genetic SRNS. Crucially, among non-genetic SRNS patients with active disease, anti-nephrin positivity was identified in 13/74 (18%) who subsequently responded to intensified immunosuppression, compared to 0/17 with multidrug-resistant disease—suggesting that the antibody may function not only as a marker of disease activity but as a predictor of immunosuppression responsiveness.

Main findings of each of these studies are shown in Table S1 (Supplementary Data).

### 3.2. Urinary Markers

Seven studies [18,19,20,22,23,41,42] researching urinary markers in relation to INS were included.

Choudhary et al. (2020) [18] measured urinary vitamin D–binding protein (VDBP) and neutrophil gelatinase-associated lipocalin (NGAL) in children with idiopathic NS (28 SRNS, 28 SSNS, plus 28 healthy controls). Mean uVDBP and uNGAL levels were markedly elevated in SRNS compared to SSNS and controls (e.g., uNGAL ~28.4 ng/mL in SRNS vs. 8.9 in SSNS vs. 6.8 in controls; *p* < 0.001). On ROC analysis, uVDBP and uNGAL discriminated SRNS from SSNS with AUC of 0.897 and 0.901, respectively, with uNGAL showing the highest likelihood ratio of the three markers tested (8.00 vs. 3.83 for uVDBP). At optimal cut-offs of ~303.8 ng/mL for uVDBP and 13.1 ng/mL for uNGAL, sensitivity was 82–86% and specificity 78–89% for identifying steroid resistance.

Urinary NGAL was also evaluated as a prognostic marker at first presentation of NS. In a prospective study of 79 children [19] (first episode, prior to steroids), baseline uNGAL < 10 ng/mL was strongly associated with steroid responsiveness by 8 weeks (*p* < 0.001). All 63 children with uNGAL ≤ 10 ng/mL achieved remission on standard prednisone, whereas 7 of 16 with uNGAL > 10 ng/mL were ultimately SRNS (the remaining 9 responded as intermediate or late responders between weeks 2 and 8). Thus, an initial uNGAL under 10 ng/mL had 100% negative predictive value for steroid resistance in this cohort.

Using a flow cytometry approach, Hidayati et al. (2023) [22] demonstrated elevated levels of podocyte-derived microparticles in pediatric NS. Urinary excretion of podocyte proteins (nephrin, podocin, and podocalyxin) in microparticle form was significantly higher in 33 children with active NS (initial onset or relapse) compared to 22 healthy controls. These urinary podocyte markers discriminated NS patients from controls with good accuracy (ROC AUC 0.895 for nephrin-MP, 0.849 for podocin, 0.728 for podocalyxin). Additionally, microparticle levels correlated positively with proteinuria severity (urine protein–creatinine ratio), with nephrin-MP showing the strongest correlation (*r* ≈ 0.70, *p* < 0.001).

Hooman et al. (2022) [20] evaluated urinary CD80 (B7-1) excretion in 51 children stratified by histopathology and steroid response. They found uCD80/Cr was dramatically higher in minimal change disease during relapse (median ~206 ng per g Cr) compared to MCD in remission (17 ng/g; *p* = 0.003), whereas FSGS cases showed low uCD80 in both relapse and remission (no significant change). Accordingly, relapsing MCD (steroid sensitive) had much greater uCD80 than relapsing FSGS (206 vs. 22.5 ng/g; *p* = 0.003). Similarly, steroid-sensitive NS patients in relapse exhibited the highest uCD80 levels (median ~402 ng/g), significantly exceeding uCD80 in SRNS relapse (124 ng/g; *p* = 0.001). In contrast, CD80 levels in remission were uniformly low and did not differ between SSNS and SRNS (~17–18 ng/g).

Li et al. (2022) [41] explored urinary sediment gene expression profiles in NS using a two-phase approach. In the discovery phase, mRNA levels of 770 immune-related genes were profiled using a NanoString nCounter platform in urinary sediment samples from six children (three SSNS and three SRNS), identifying 70 differentially expressed genes between the two groups. From these, nine candidates were selected for RT-PCR validation in an expanded cohort of 129 children with INS (94 SSNS, 35 SRNS). Two immune-related genes were identified as differentially expressed by steroid response: CREBBP (CREB-binding protein) mRNA was significantly elevated in SRNS compared to SSNS (*p* = 0.02), while CYBA (encoding p22^phox^) was significantly lower in SRNS (*p* = 0.01). Individually, these transcripts showed modest ability to distinguish SRNS from SSNS (AUC 0.655 for CREBBP, 0.669 for CYBA). At defined cut-offs, CREBBP had high sensitivity (83.3%) but limited specificity (49.4%), whereas CYBA was less sensitive (58.3%) but more specific (83.1%). A combined CREBBP + CYBA marker improved overall diagnostic performance (AUC 0.666, sensitivity 63.9%, specificity 76.4%) for predicting steroid resistance. The small discovery-phase sample size and the modest AUC values of both individual and combined markers reflect the preliminary nature of these findings, which await independent replication.

Lodeweyckx et al. (2021) [42] investigated urinary epidermal growth factor (uEGF) as a marker of tubular health in 98 pediatric NS or persistent proteinuria patients (various biopsy diagnoses), with normal GFR. Overall, children with nephrotic-range disease had significantly lower uEGF excretion than healthy peers (~46% reduction in uEGF/Cr; *p* < 0.001). Notably, baseline uEGF/Cr was relatively higher in MCD than in other glomerulopathies: in the absence of ACE inhibitor, MCD patients had a 57% higher uEGF/Cr than those with FSGS, IgA nephropathy, or other lesions (*p* = 0.026). Conversely, children with FSGS/other NS had more severely depressed uEGF levels (~61% below normal). Introduction of an ACE inhibitor produced a significant rise in uEGF output in FSGS/other glomerulopathies (+78% uEGF/Cr under ACEi) but no effect in MCD patients. Thus, MCD exhibited a distinct uEGF profile—higher relative uEGF and lack of ACEi response—compared to more injurious etiologies. It should be noted that the study cohort extended beyond classical INS, encompassing children with persistent non-nephrotic proteinuria and various biopsy diagnoses including IgA nephropathy; furthermore, urine samples were collected during remission under ongoing immunosuppressive therapy rather than at active disease presentation, which limits direct comparability with other biomarker studies in this review.

Thy et al. (2021) [23] measured soluble urokinase plasminogen activator receptor in urine (suPAR/Cr) in 92 children (49 new-onset NS, 43 relapses). Urinary suPAR levels were significantly elevated in active relapse cases (median ~628 pg/μmol Cr) compared to initial-onset NS (~509) and to healthy controls (~248; *p* = 0.001). In the initial NS group, children who were steroid resistant showed higher suPAR/Cr after treatment than those who were steroid sensitive, but this difference did not reach significance. However, among relapsing patients, suPAR/Cr was significantly higher in those with steroid-resistant courses than in relapsing SSNS (*p* = 0.02). A suPAR/Cr threshold of 950 pg/μmol distinguished SRNS with ~73.9% sensitivity and 89.5% specificity (AUC ~0.70, *p* < 0.001). Main findings of each of these studies are shown in Table S2 (Supplementary Data).

### 3.3. Cytokines and Chemokines

Four studies evaluated circulating cytokines and chemokines in children with idiopathic nephrotic syndrome (INS), focusing on their associations with disease activity, relapse, and steroid responsiveness.

Nickavar et al. (2020) [43] examined 48 children with INS (21 SSNS, 27 SRNS) and 19 healthy controls. Serum IL-1β, IL-2, IL-6, and IL-8 were significantly higher in INS versus controls, while IL-13 and IL-18 were lower. Among disease subgroups, SSNS showed higher IL-1β, IL-6, and IL-8 than SRNS. ROC analysis identified IL-8 as the most accurate marker for distinguishing INS from healthy controls (AUC 0.890, sensitivity 87.2%, specificity 94.7%), while for steroid response prediction, IL-1β achieved the highest AUC (0.819), with IL-8 showing moderate accuracy (AUC 0.681).

Agrawal et al. (2021) [24] analyzed 40 children with new-onset NS (26 SSNS, 14 SRNS) with paired plasma samples before and after ~7 weeks of glucocorticoid (GC) therapy. Multiplex bead-based assays quantified 27 cytokines. Pretreatment, 13 cytokines differed between SSNS and SRNS; logistic regression identified a 3-cytokine panel (IL-7, IL-9, MCP-1) with ROC 0.846, sensitivity 0.643, specificity 0.846 for predicting SRNS. GC treatment decreased IFN-γ, TNF-α, IL-7, IL-13, and IL-5 in both groups, but relative differences between SSNS and SRNS persisted.

Kopač et al. (2025) [44] performed a prospective longitudinal study in 18 Slovenian children with INS (MCD predominant) using Olink Target 48 panels across disease onset/relapse, remission on corticosteroids, and remission after steroid withdrawal. Significant plasma differences were found for CSF1, IL-4, FLT3LG, CCL19, and MMP12 at onset/relapse versus remission; CSF1 and IL-17F distinguished onset/relapse versus post-treatment remission; and CCL19, MMP12, and CCL13 rose again after steroid discontinuation. These markers tracked closely with disease activity.

Gowtham et al. (2024) [25] enrolled 112 children (1–14 years) with idiopathic NS and measured serum TNF-α at presentation and at remission or day 42 if steroid resistant. Median TNF-α fell significantly overall from 7.5 (IQR 3.5–12.1) to 5.25 (IQR 1.62–8.8) pg/mL (*p* = 0.01). By phenotype, significant reductions were observed in first-episode NS (3.98 → 1.88 pg/mL, *p* = 0.04) and IFRNS (9.6 → 5.94 pg/mL, *p* = 0.04), while SRNS showed a non-significant rise from 6.59 to 9.02 pg/mL (*p* = 0.27). Notably, SDNS had the highest baseline TNF-α of all subgroups (median 10.51 pg/mL), which the authors attributed to cumulative podocyte injury from recurrent proteinuria episodes. Higher baseline TNF-α was associated with a longer time to remission, with each 5.47 pg/mL increment at presentation corresponding to approximately one additional day of steroid therapy (*p* = 0.037), supporting a relationship between inflammatory burden and steroid responsiveness. The SRNS subgroup comprised only four patients, limiting conclusions about steroid resistance. Main findings of these studies are also shown in Table S3 (Supplementary Data).

### 3.4. Immune Cell Signatures

Seven studies profiled peripheral blood immune cells in children with idiopathic NS using flow cytometry, mass cytometry (CyTOF), and routine hematological indices, collectively describing broad immune dysregulation across T cells, B cells, NK cells, and monocytes, with phenotype-specific patterns that distinguish steroid-sensitive from steroid-resistant and relapsing disease—and, in the post-rituximab setting, identify children at risk of relapse.

Ye et al. (2021) [45] profiled peripheral blood mononuclear cells in 85 children (59 SSNS, 26 SRNS) and 30 controls before and after corticosteroids. Both phenotypes showed expanded effector CD8^+^ T cells and reduced non-classical monocytes, but B-cell expansion (total B cells and plasmablasts) together with depletion of activated Tregs was particularly characteristic of the steroid-sensitive phenotype, alongside an NK subset shift toward CD56^hi^ cells.

Chen et al. (2021) [21] evaluated 285 children (187 SSNS, 98 SDNS/FRNS) and 50 controls at admission, prior to steroids. Both NS groups had reduced CD3^+^, CD4^+^, and CD4^+^/CD8^+^ ratios versus controls, with the CD4^+^/CD8^+^ ratio lowest in SDNS/FRNS. Urinary CD80 was higher in SSNS than SDNS/FRNS, consistent with its proposed role as a marker of steroid-sensitive/MCD disease, while a reduced IgG/IgM ratio (<3) and higher hs-CRP and IL-6 tracked the relapsing/dependent phenotype.

Deng et al. (2023) [28] retrospectively analyzed lymphocyte subsets in 204 children across SSNS, SRNS, and SDNS/FRNS with 19 controls. B-cell kinetics diverged by phenotype—CD19^+^ B cells were elevated at onset in SRNS but rose in remission in SDNS/FRNS. ROC analysis identified CD19^+^ B cells as a predictor of SRNS (AUC 0.718; cut-off 17.46%/L; sensitivity 71.2%, specificity 70.8%), and a CD8^+^ T/CD19^+^ B ratio cut-off of 2.22 distinguished active SDNS (AUC 0.805). NK (CD56^+^CD16^+^) cells were reduced across all groups, echoing Ye et al.

Fribourg et al. (2021) [27] used time-of-flight mass cytometry to track immune reconstitution in 30 children with SDNS after B-cell-depleting therapy (rituximab or ofatumumab) and subsequent immunosuppression withdrawal; 17 relapsed and 13 remained stable. Although total B-cell depletion was comparable between groups, five class-switched B-cell subsets were significantly more abundant in relapsers at both baseline and relapse (*p* < 0.05), establishing class-switched B-cell reconstitution as the dominant immune signal of relapse risk and a rationale for monitoring these subsets to guide retreatment timing.

Jamee et al. (2022) [46] assessed neutrophil-to-lymphocyte and platelet-to-lymphocyte ratios in 50 children spanning all phenotypes; neither changed significantly with steroid therapy nor associated with steroid-response category.

Riganati et al. (2024) [26] enrolled 44 children with new-onset INS, sampled at diagnosis and after 3 months of prednisone, with 12-month follow-up. At onset, B-cell subsets were elevated and Tregs reduced relative to controls. Among the 40 SSNS patients, relapsers had higher baseline transitional and memory B cells; unswitched memory B cells retained independent predictive value after age adjustment (OR 3.6), with a >1.5% cut-off yielding an AUC of 0.79 for relapse within 12 months.

Al-Aubodah et al. (2023) [39] provided a comprehensive characterization of the B-cell compartment in childhood INS, combining single-cell RNA sequencing with multiparameter flow cytometry across a longitudinal cohort of 31 children (active INS, glucocorticoid-induced remission, rituximab-maintained remission, and post-rituximab relapse) alongside 24 healthy controls. Perturbation of the B-cell transcriptional landscape was the dominant immune abnormality in active disease, characterized by an extrafollicular response with expansion of CD21^low^/CXCR5^−^/T-bet^+^/CD11c^+^ atypical B cells and short-lived antibody-secreting cells. Glucocorticoids reduced antibody-secreting cells alone, whereas rituximab ablated the full spectrum of INS-associated B cells, and a nascent resurgence of marginal-zone-like B cells marked post-rituximab relapse. These findings mechanistically link the B-cell populations targeted by rituximab to the generation of podocyte-directed autoantibodies, bridging the autoantibody and immune-cells.

An overview of these studies is provided in Table S4 (Supplementary Data).

### 3.5. Further Biomarkers

Beyond autoantibodies, urinary markers, cytokines, and immune cell signatures, a heterogeneous group of studies has explored circulating proteins, metabolites, lipid profiles, extracellular vesicles, and microRNAs as candidate biomarkers in pediatric INS. While largely exploratory, these investigations provide mechanistic insights into disease activity, steroid responsiveness, and relapse biology.

In a companion plasma proteomic study from the same consortium we mentioned earlier, Agrawal et al. (2020) [40] analyzed paired pre- and post-treatment samples using a discovery cohort of 15 paired samples followed by validation in 37 patients (24 SSNS, 13 SRNS). A three-protein panel—vitamin D-binding protein, adiponectin, and matrix metalloproteinase-2—predicted steroid resistance at presentation with an AUC of 0.78, while hemopexin and apolipoprotein L1 further distinguished steroid-sensitive from steroid-resistant disease. This signature independently converges on markers identified elsewhere in this review, including urinary vitamin D-binding protein [18] and hemopexin [30].

Aksu et al. (2024) [29] investigated heat shock protein 70 (HSP70), a stress-response chaperone implicated in podocyte injury, in a prospective cohort of 36 children with SSNS compared to 35 healthy controls, with serial sampling at days 0, 15, 30, and 90 after starting prednisolone. At baseline, urinary HSP70 (uHSP70) and uHSP70/creatinine ratio were significantly elevated in SSNS compared to controls (*p* = 0.001 and *p* = 0.034, respectively), while serum HSP70 was paradoxically lower (*p* = 0.002). Over the course of treatment, uHSP70 declined significantly (Friedman *p* < 0.0001), and baseline uHSP70 correlated positively with proteinuria (r = 0.347, *p* = 0.048) and inversely with serum albumin (r = −0.352, *p* = 0.045), suggesting it reflects glomerular injury burden. No differences in HSP70 levels were observed between frequent and infrequent relapsers at any timepoint, limiting its prognostic utility within the SSNS phenotype.

Pukajło-Marczyk et al. (2021) [30] evaluated hemopexin (Hpx), an acute-phase heme-binding protein, in 51 children with INS—20 at first onset and 31 with relapses—alongside 18 healthy controls, using paired relapse/remission sampling. Both serum and urinary hemopexin were markedly elevated during relapse compared to controls and declined significantly at remission (all *p* < 0.0001), though they remained above control levels even in remission. Notably, urinary hemopexin in remission was significantly higher in children with prior relapses than in those experiencing their first episode (*p* = 0.006), suggesting a cumulative pattern of renal stress. Urinary hemopexin also differed by treatment intensity: levels were higher during relapse and lower in remission in patients receiving steroids alone compared to those on combined immunosuppression, while serum hemopexin was unaffected by treatment regimen.

Udagawa et al. (2021) [31] examined serum IgM as a predictor of steroid resistance in a multicenter retrospective registry of 80 children with idiopathic NS, of whom 13 were SRNS. At diagnosis, lower serum IgM—analyzed by tertiles—was independently associated with SRNS after adjustment for confounders (OR 6.94, 95% CI 1.12–43.11). Neither the selectivity index nor the total protein-to-albumin ratio showed a significant association, suggesting that among readily available laboratory parameters, IgM may carry a specific prognostic signal for steroid resistance.

Wen et al. (2023) [32] assessed angiopoietin-like protein 3 (ANGPTL3), a circulating inhibitor of lipoprotein lipase implicated in NS-associated dyslipidemia, in 200 children with NS and 80 healthy controls. Both serum ANGPTL3 and urinary ANGPTL3 normalized to creatinine were significantly elevated in NS compared to controls (both *p* < 0.0001). Among proteinuric NS patients (*n* = 126), urinary ANGPTL3/creatinine demonstrated excellent diagnostic performance (AUC 0.975, sensitivity 94.4%, specificity 92.5% at a cut-off of 0.0101 ng/g), outperforming serum ANGPTL3 alone (AUC 0.800), with a combined AUC of 0.989. Both markers correlated positively with proteinuria and lipid parameters and inversely with serum albumin, consistent with ANGPTL3 upregulation as a downstream consequence of hypoalbuminemia and nephrotic dyslipidemia.

Gooding et al. (2020) [33] applied proton nuclear magnetic resonance (^1^H-NMR) metabolomics to plasma samples from 45 children (30 SSNS, 15 SRNS) collected before and approximately seven weeks after glucocorticoid therapy, generating 86 paired samples. Stepwise logistic regression identified creatinine and glutamine as candidate predictors of steroid resistance (OR 1.01 each), with malonate emerging as an additional discriminator in children over three years of age (OR 0.94). Paired analyses highlighted further candidate metabolites—including lipoproteins, adipate, pyruvate, creatine, glucose, and sn-glycerophosphocholine—suggesting perturbations in energy metabolism and redox homeostasis as correlates of steroid resistance, though none have yet been externally validated.

Hu et al., (2023) [34] performed untargeted metabolomic profiling using UHPLC-QTOF-MS in serum samples from 27 treatment-naïve SSNS children compared to 56 healthy controls, with parallel urine profiling in 17 SSNS and 24 controls, followed by targeted UPLC-MS/MS validation in an independent cohort. Serum metabolomics yielded clear separation between SSNS and controls, while urine profiles failed to discriminate the two groups, supporting serum as the superior matrix for early disease detection. Among 194 significantly altered serum metabolites, eight were selected as candidate biomarkers (AUC = 1, log_2_FC > 5): D-mannitol, dulcitol, D-sorbitol, and XMP were elevated in SSNS, while NADPH, NAD, bilirubin, and α-KG were reduced. Causal inference modeling confirmed that all eight exerted a causal effect on SSNS status, with the most significantly altered pathways implicating amino acid metabolism and the TCA cycle.

Cricri et al. (2024) [35] conducted a comprehensive flow cytometric characterization of extracellular vesicle (EV) surface markers in serum and urine from 105 children with INS across disease states—onset, relapse, persistent proteinuria, and remission—alongside 19 healthy controls, profiling 37 EV surface antigens. Urinary tetraspanins were elevated during active disease and correlated with proteinuria severity. Unsupervised clustering identified an 8-marker EV signature spanning immune, angiogenic, and adhesion pathways, with CD41b, CD29, and CD105 providing the best separation between active INS and healthy controls. Combining serum and urinary EVs from the same patient improved disease staging accuracy, while urinary CD19, CD44, and CD8 markers classified patients by steroid sensitivity, supporting EVs as a multidimensional, non-invasive biomarker platform.

Turolo et al., 2021 [36] characterized the peripheral blood fatty acid profile in 47 children with INS in stable remission compared to 47 matched healthy controls using gas chromatography. Despite being in remission, INS patients showed persistently elevated proportions of total polyunsaturated fatty acids (PUFA; 40.17% vs. 37.91%) and omega-6 fatty acids (36.95% vs. 34.79%), driven principally by higher linoleic acid (23.01% vs. 21.55%, *p* = 0.003) and arachidonic acid (10.37% vs. 9.65%, *p* = 0.01), alongside reduced saturated and monounsaturated fatty acid fractions. The persistence of a pro-inflammatory omega-6-enriched lipid milieu during clinical remission raises the hypothesis that residual lipid dysregulation may contribute to relapse susceptibility, independent of proteinuria.

Sinha et al., 2025 [37] conducted whole-blood microRNA microarray profiling of 2549 miRNAs in 12 SSNS children (at onset or relapse, off immunosuppression), 24 SRNS patients (at diagnosis, on prednisolone), and 12 healthy controls. Sixty-two miRNAs were differentially expressed in SSNS and 12 in SRNS relative to controls, with 76 miRNAs differing between the two phenotypes—26 unique to SSNS and 11 unique to SRNS; miR-125b was the only finding consistent with prior literature, showing upregulation in SRNS. Target-gene enrichment identified 79 disease-relevant genes among the miRNA targets, with the most significantly enriched biological processes including NF-κB signaling, apoptosis regulation, and gene silencing by RNA, implicating pathways relevant to both podocyte integrity and immune dysregulation. The authors acknowledged the pilot nature and limited sample size as key constraints, calling for validation in larger independent cohorts. Table S5 (Supplementary Data) provides an overview of these studies.

## 4. Discussion and Clinical Implications

The 34 studies reviewed here collectively challenge the long-standing concept of childhood idiopathic nephrotic syndrome (INS) as a uniform, empirically managed condition. Across independent cohorts and diverse methodological approaches, a consistent picture emerges: pediatric INS represents a spectrum of immune-mediated podocytopathies and genetic podocyte diseases, that are fundamentally distinct in their pathogenesis, treatment responsiveness, and long-term prognosis. This reconceptualization has direct clinical implications, moving the field from empirical corticosteroid trials toward a biology-guided classification. Among the candidate biomarkers reviewed, anti-nephrin autoantibodies provide the most compelling evidence that an identifiable immune mechanism drives a substantial subset of pediatric INS, and they are examined in detail below; this mechanistic primacy should not, however, be conflated with clinical readiness, and the sections that follow deliberately weigh anti-nephrin against the more modest but more immediately deployable markers.

### 4.1. Anti-Nephrin Autoantibodies: Converging Evidence for an Autoimmune Podocytopathy

Evidence has accumulated rapidly across independent cohorts and methodological platforms, each study adding a distinct dimension to the clinical picture. Watts et al. [14] first documented anti-nephrin IgG in 29% of minimal change disease patients during active relapse, with complete disappearance in paired remission samples and functional nephrin binding confirmed by immunoprecipitation. Hengel et al. [7] subsequently extended these findings to a multicenter pediatric cohort of 182 children, reporting positivity in 52% overall and in up to 90% of those actively nephrotic without immunosuppression—with antibody levels tracking longitudinally with proteinuria. Raglianti et al. [15] added a histological dimension, identifying IgG deposits co-localizing with nephrin along the slit diaphragm in 30% of MCD and 25% of FSGS pediatric biopsies, with antibody-positive patients achieving remission in 92% of cases compared to only 20% of antibody-negative peers, and none progressing to kidney failure. Most recently, the PodoNet multicenter study [16] mapped seroprevalence across the full phenotypic spectrum: 68% in SSNS, 28% in SDNS, 14% in non-genetic SRNS, and near-zero in genetic SRNS. Crucially, within non-genetic SRNS, positivity was confined exclusively to patients who subsequently responded to intensified immunosuppression—identifying anti-nephrin as a predictor not only of immune pathogenesis but of treatment responsiveness.

The breadth of the autoantibody phenomenon extends beyond nephrin alone. Ye et al. [17] identified seven podocyte-targeting autoantibodies in two-thirds of a large INS cohort, with anti-vinculin being the most frequent, and titers correlating dynamically with disease activity. Together, these findings support the reframing of a substantial proportion of pediatric INS as *autoimmune podocytopathy*—a term with direct therapeutic implications, drawing a parallel to anti-PLA_2_R membranous nephropathy, where serology now guides diagnosis and treatment monitoring as a matter of routine practice.

### 4.2. Anti-Nephrin in Perspective: What the Antibody Cannot Do

The consistency of the evidence above should not be mistaken for clinical readiness, and four constraints deserve emphasis before anti-nephrin is allowed to dominate the biomarker conversation. First, sensitivity is incomplete even under ideal conditions: in the most favorable sampling window—active, untreated, nephrotic disease assessed in a research laboratory—10–20% of children with presumed immune-mediated INS are seronegative [7], so a negative result can never exclude an immune mechanism and cannot, on its own, redirect a child toward genetic investigation, for example. Second, seropositivity is strongly state- and treatment-dependent, falling to 28% in SDNS and 14% in non-genetic SRNS [16]; this is a serious practical inversion, because the phenotypes in which a prognostic marker would be most valuable are precisely those in which the antibody is least often detectable, while the phenotype in which it is most often detected (SSNS, 68%) is the one in which the steroid trial already delivers the answer within four weeks and at negligible cost. Third, no anti-nephrin assay is currently available outside research settings, whereas several of the alternative markers discussed below can be measured today on platforms that most pediatric nephrology units already own. Fourth, the autoantibody landscape is broader than nephrin alone: Ye et al. [17] identified seven podocyte-targeting autoantibodies, anti-vinculin being detected in 56% of cases, and Raglianti et al. [15] found that roughly a fifth of biopsies with slit-diaphragm IgG deposition showed no nephrin co-localization, implying additional and as yet uncharacterized target antigens. Anti-nephrin is therefore best understood as the first and best-characterized member of a family of podocyte autoantibodies, and as the strongest available mechanistic evidence that a substantial share of pediatric INS is autoimmune—rather than as a test that is ready to displace, or even substantially modify, current practice.

### 4.3. Challenges for Clinical Translation of Anti-Nephrin Testing

Despite the strength and consistency of the above evidence, significant barriers currently prevent anti-nephrin testing from entering everyday clinical practice. These challenges span assay methodology, biological complexity, healthcare infrastructure, and regulatory pathway, and must be addressed before this biomarker can fulfill its clinical promise.

Assay heterogeneity is the most immediate obstacle. Across the studies reviewed, anti-nephrin detection was performed by immunoprecipitation (qualitative), hybrid IP–ELISA (semi-quantitative), cell-based assays, and biopsy immunofluorescence—four methodologically distinct approaches with no established concordance data. No standardized, commercially available assay currently exists. This means that results from different centers are not directly comparable, quantitative thresholds cannot be harmonized across platforms, and there is no validated reference standard against which new assay formats can be calibrated. The analogy with anti-PLA_2_R testing is instructive: it took over a decade from initial discovery to the availability of a CE-marked clinical ELISA, and standardization of anti-PLA_2_R quantification remains incomplete. Anti-nephrin is at an earlier stage of that trajectory.

Incomplete sensitivity presents a second challenge. Even under the most favorable conditions—active untreated disease, in a dedicated research setting—10–20% of children with presumed immune-mediated INS are seronegative. The proportion is substantially higher in immunosuppressed patients, in remission, or in the SDNS phenotype, reflecting the known dependence of circulating autoantibody levels on disease state and treatment. This means that a negative result cannot exclude immune-mediated disease, complicating the clinical interpretation of routine testing in treated patients. The optimal timing of sampling—before corticosteroid initiation at first presentation, or during relapses while off immunosuppression—is yet to be formally validated prospectively. Serial monitoring has been proposed but protocols are missing.

Access and infrastructure represent a further constraint, particularly relevant in the settings where pediatric nephrotic syndrome is most prevalent—South and Southeast Asia, sub-Saharan Africa, and Latin America. Immunoprecipitation-based assays require specialized laboratory infrastructure and experienced personnel that are not available in most centers caring for children with INS globally. Even in well-resourced settings, turnaround time for research-grade assays is incompatible with acute clinical decision making. Until a robust point-of-care or centralized laboratory ELISA is validated and commercially distributed, the practical reach of anti-nephrin testing will remain limited to specialist or academic centers.

Finally, the regulatory and validation pathway remains largely uncharted. No prospective randomized trial has yet evaluated whether anti-nephrin-guided treatment allocation—for example, early rituximab in seropositive SRNS, or accelerated genetic testing in seronegative SRNS—improves clinical outcomes compared to standard care. The observational data, while compelling, carry the limitations of retrospective ascertainment, referral bias, and non-standardized treatment protocols across centers. Before anti-nephrin testing can be embedded in clinical guidelines alongside existing KDIGO and IPNA recommendations, it requires prospective multicenter validation in patient populations, with pre-defined testing protocols and outcome definitions.

### 4.4. Complementary Biomarkers: Toward a Multi-Analyte Framework

Beyond autoantibodies, converging evidence from urinary, cytokine, and immune cell studies supports a multi-analyte approach to biomarker-guided management of pediatric INS. Urinary NGAL has shown the most consistent performance, with two independent cohorts reporting its ability to predict steroid resistance at disease onset—including a 100% negative predictive value for SRNS at a threshold below 10 ng/mL [18,19]. Urinary CD80 provides complementary specificity for the MCD/steroid-sensitive phenotype [20], while podocyte-derived microparticles track active podocyte injury in real time [22]. Together, these non-invasive markers could constitute a diagnostic panel at first presentation, reducing the interval before steroid resistance is recognized and genetic investigation initiated.

Immune cell signatures add prognostic granularity, particularly in the relapse-prone patient. Elevated unswitched memory B cells at diagnosis predicted 12-month relapse in newly diagnosed SSNS with an AUC of 0.79 [26], while post-rituximab reconstitution of class-switched memory B cells identified relapsers before clinical relapse occurred [27]. These findings align with the known clinical efficacy of B-cell depletion in SDNS and FRNS and provide a biological rationale for monitoring B-cell subsets as a guide to retreatment timing. Cytokine profiles—notably the IL-7/IL-9/MCP-1 panel and dynamic TNF-α behavior—offer additional early discrimination between SSNS and SRNS and may help characterize inflammatory burden at diagnosis [24,25,43]. Exploratory approaches including metabolomics, microRNA profiling, extracellular vesicle signatures, and fatty acid analysis provide mechanistic insights into disease biology and may eventually contribute to composite biomarker panels but remain investigational at this stage.

### 4.5. Strength of the Underlying Evidence

Applying the appraisal framework described in the Methods, the evidence base reviewed here is markedly uneven. The strongest evidence (Tier A) supports the autoantibody findings and the B-cell signatures: the anti-nephrin data derive from large multicenter cohorts with phenotype-defined comparators and longitudinal corroboration of antibody titers against disease activity [7,15,16], reinforced by a large independent autoantibody screen [17]; the B-cell findings rest on a prospective longitudinal cohort with 12-month outcome ascertainment [26] and a prospective mass-cytometry study of post-depletion reconstitution [27]; and the cytokine panel derives from a multicenter consortium study with paired pre- and post-treatment sampling [24]. Findings from these studies are the most likely to replicate. A second, larger group (Tier B) comprises methodologically sound single-center studies with adequate samples and appropriate controls but no independent validation—this includes the entire urinary NGAL literature [18,19], urinary CD80 [20], podocyte microparticles [22], suPAR [23], serum IgM [31], ANGPTL3 [32], and the lymphocyte-subset series [21,28,45]; here the reported cut-offs and AUCs should be regarded as provisional until reproduced in an independent cohort. The remaining studies (Tier C) are explicitly exploratory: the metabolomic [33,34], microRNA [37], urinary transcriptomic [41], and cytokine-profiling [44] analyses are discovery-phase, high-dimensional investigations conducted in cohorts of 6 to 45 children with no validation set, in which the risk of overfitting is substantial and impressively high AUC values should be interpreted with corresponding caution. The negative findings for the neutrophil-to-lymphocyte and platelet-to-lymphocyte ratios [46] likewise come from a small cohort. Readers should therefore weight the evidence accordingly: the mechanistic case for an autoimmune podocytopathy and the prognostic value of B-cell subsets are the most securely established conclusions of this review, whereas the specific quantitative thresholds proposed for individual urinary and metabolic markers remain to be confirmed.

### 4.6. Barriers to Implementation and Near-Term Clinical Prospects

The obstacles to clinical use are not unique to anti-nephrin; they recur across every biomarker class reviewed here. Assay standardization is largely absent—urinary NGAL, CD80, and suPAR have each been measured with different immunoassays without cross-platform calibration, so cut-offs cannot be transferred between laboratories. Decision thresholds are correspondingly unvalidated, and no interventional trial has yet tested whether biomarker-guided management improves outcomes, which is the evidence guideline committees will require. Cost and infrastructure impose a further constraint, since INS is most prevalent in regions where mass spectrometry, mass cytometry, or immunoprecipitation are not routinely available. Against this background, the candidates differ in proximity to the clinic. Those likeliest to be used in the near term require no new technology: urinary NGAL is already measured on automated analyzers and is supported by the most internally consistent data in this review; peripheral B-cell subset monitoring requires only flow cytometry already used where rituximab is given; and serum IgM and standard lymphocyte subsets are inexpensive and near universal. Urinary CD80 and anti-nephrin serology are credible medium-term prospects contingent on a standardized, commercially distributed assay. Metabolomic, microRNA, and extracellular-vesicle signatures remain investigational, valuable for understanding disease biology but resting on small discovery cohorts. A realistic near-term vision is therefore not a single test replacing the steroid trial, but a modest panel—a urinary injury marker and a B-cell profile at presentation—that stratifies risk early enough to shorten the interval before steroid resistance is recognized, with autoantibody serology added as validated assays become available.

### 4.7. Limitations of the Current Evidence Base

Several methodological limitations constrain the clinical translation of findings across all biomarker categories reviewed. The majority of included studies are small, single-center investigations with limited sample sizes, restricting both statistical power and general applicability. Assay platforms vary widely, with no standardized commercial alternatives available for most candidate markers. Cross-sectional and retrospective designs predominate, limiting the ability to establish predictive relationships independent of concurrent clinical variables. Geographic representation is skewed toward East and South Asian populations, which may not reflect the full spectrum of immune and genetic architecture in other ethnic groups. Addressing these limitations will require prospective multicenter studies, consortium-level data sharing, and the development of standardized, validated assay protocols across biomarker classes.

## 5. Conclusions and Future Directions

The trajectory of evidence points toward a future in which pediatric INS is managed according to a measurable biological “signature” rather than empirical treatment response. In the near term, the most actionable priorities are standardization of anti-nephrin assay formats with multicenter concordance studies, prospective validation of urinary NGAL and CD80 as first-presentation predictors of steroid resistance in diverse populations, and exploratory clinical trials of biomarker-guided treatment allocation—particularly early B-cell depletion in seropositive SDNS and expedited genetic evaluation in seronegative SRNS. In the longer term, integration of multi-omics approaches—combining autoantibody serology, immune cell profiling, urinary proteomics, and metabolomics—may yield composite biomarker signatures with sufficient sensitivity and specificity to replace or substantially reduce reliance on the current empirical steroid trial. Such an approach would align the management of pediatric INS with the precision medicine paradigm already emerging in other immune-mediated glomerular diseases.

## Figures and Tables

**Table 1:** IPNA and KDIGO guidelines on nephrotic syndrome.

Topic	IPNA (2020 SRNS + 2022 SSNS)	KDIGO 2025
Idiopathic nephrotic syndrome definition	Triad of nephrotic-range proteinuria (≥40 mg/m^2^/h or uPCR > 200 mg/mmol ≈ >2 g/g), hypoalbuminemia (usually <30 g/L), and edema; edema can be absent if biochemical criteria met.	Same clinical triad
SSNS definition and treatment protocol	SSNS: complete remission within 4 weeks of standard prednisone. Initial episode steroids: total 8 or 12 weeks—e.g., 4 weeks of 60 mg/m^2^/day, max 60 mg, then 4 weeks of 40 mg/m^2^ in alternate days, or 6 + 6 with same dosage.	Same recommendations—no benefit beyond 12 weeks; favor minimizing exposure.
FRNS definition	Frequent relapser: ≥2 relapses in 6 months or ≥4 in 12 months.	Same definition.
SDNS definition	Steroid-dependent: relapse during taper or within 14 days of stopping steroids (typically two consecutive relapses in this window).	Same definition.
SRNS definition and confirmation	SRNS: no complete remission after 4 weeks of daily prednisone; confirmation period weeks 4–6 includes IV methylprednisolone pulses ± RAAS blockade; biopsy/genetics during this window.	SRNS: no remission at 4 weeks; explicit 4–6 week confirmation; defines response phenotypes (CNI-responsive/resistant; multidrug-resistant).
Kidney biopsy indication	At onset: only with atypical features (gross hematuria, low C3, unexplained AKI, sustained HTN, vasculitic signs, etc.). All SRNS: biopsy at 4–6 weeks.	Same recommendations.
Genetic testing	Primary SRNS: recommend testing for all (prioritize familial/syndromic/early-onset); consider before biopsy when feasible; provide genetic counseling. SSNS: consider if atypical/very early onset.	Same recommendations.
SRNS treatment (post-steroid)	First-line: CNI (cyclosporine or tacrolimus) + RAAS blockade; taper/stop prednisone by 6 months. Subsequent options: rituximab, MMF, cyclophosphamide (context-dependent).	Same recommendations. Places stronger emphasis on early biopsy/genetics guiding therapy.
CNI targets and dosing for SRNS	CsA: start at 5–6 mg/kg/day, trough levels of 80–120 ng/mL. Tacrolimus: start at 0.1–0.2 mg/kg/day, trough 4–8 ng/mL. Monitor levels (weekly during titration → q1–3 mo); reduce/stop if eGFR falls (<30 mL/min/1.73 m^2^).	Same recommendations, but less prescriptive on exact target levels.
How long to try CNI before declaring failure	If no partial remission by 6 months → declare CNI-resistant and switch. If complete remission, consider stopping at 12–24 months or switch to MMF to limit nephrotoxicity.	Aligns conceptually: classify CNI-resistant at 6 months without partial remission; CNI-responsive if partial at 6 months and/or complete by 12 months.
FRNS/SDNS–steroid-sparing sequence	Emphasizes levamisole (esp. FRNS), with context-driven use of cyclophosphamide, MMF, CNIs, rituximab based on phenotype/toxicity/resources.	Suggests levamisole or cyclophosphamide often preferred for FRNS; MMF, rituximab, CNIs favored for SDNS; highlights tailoring to toxicity, access, and preferences.
Vitamin D/calcium and gastroprotection	Routine bone-health monitoring in FR/SD NS; supplement vitamin D/calcium per levels; minimize steroid exposure.	No routine PPI unless risk factors; vitamin D/calcium not routinely required if levels normal; consider in FR/SDNS or deficiency.
Thrombosis prophylaxis	Not routine; consider LMWH/oral agents if prior VTE or high-risk (CVC, thrombophilia, hospitalization, dehydration).	Same risk-based approach.

Abbreviations: INS = idiopathic nephrotic syndrome; uPCR = urine protein-to-creatinine ratio. SSNS = steroid-sensitive nephrotic syndrome; SRNS = steroid-resistant nephrotic syndrome; FRNS = frequently relapsing nephrotic syndrome; SDNS = steroid-dependent nephrotic syndrome; CNI = calcineurin inhibitor (cyclosporine or tacrolimus); CsA = cyclosporine A; TAC = tacrolimus; MMF = mycophenolate mofetil; RAAS = renin–angiotensin–aldosterone system; LMWH = low-molecular-weight heparin; VTE = venous thromboembolism; PPI = proton pump inhibitor; CVC = central venous catheter.

**Table 2:** Summary of the most clinically relevant candidate biomarkers in pediatric idiopathic nephrotic syndrome.

Biomarker	Sample	Key Finding/Performance	Clinical Role	Ref(s)
Anti-nephrin IgG	Serum	Seroprevalence 68/28/14/2% (SSNS/SDNS/non-genetic SRNS/genetic SRNS); positivity in non-genetic SRNS predicts response to intensified immunosuppression	Diagnosis; predicts IS response	[7,14,15,16]
Other podocyte autoantibodies (e.g., anti-vinculin)	Serum	≥1 autoantibody in 66%; titres track disease activity	Diagnosis	[17]
Urinary NGAL (±VDBP)	Urine	AUC ≈ 0.90 for SRNS; uNGAL < 10 ng/mL = 100% NPV for SRNS at first episode	Predicts steroid resistance at onset	[18,19]
Urinary CD80	Urine	Elevated in MCD/SSNS relapse; low in FSGS/SRNS	Marks steroid-sensitive/MCD phenotype	[20,21]
Podocyte microparticles (nephrin, podocin)	Urine	AUC up to 0.90 vs. controls; correlate with proteinuria	Marks active podocyte injury	[22]
Urinary suPAR	Urine	AUC ≈ 0.70; higher in SRNS relapse	Associated with steroid resistance	[23]
Cytokine panel (IL-7/IL-9/MCP-1)	Plasma	AUC 0.85 for SRNS	Predicts steroid resistance	[24]
Serum TNF-α	Serum	Falls with remission in SSNS, highest in SDNS; higher baseline → longer time to remission	Disease-activity monitoring	[25]
Unswitched memory B cells	Blood	OR 3.6; >1.5% cut-off, AUC 0.79	Predicts 12-month relapse in SSNS	[26]
Class-switched memory B cells	Blood	More abundant in relapse after B-cell depletion	Predicts relapse post-rituximab (SDNS)	[27]
CD19^+^ B cells/CD8^+^ T:CD19^+^ B ratio	Blood	AUC 0.72 (SRNS); ratio AUC 0.81 (SDNS)	Phenotype discrimination	[28]
Exploratory: metabolomics, miRNA, EVs, ANGPTL3, HSP70, fatty acids	Serum/Urine	Investigational; some high AUCs (e.g., uANGPTL3 AUC 0.98)	Hypothesis-generating	[29,30,31,32,33,34,35,36,37]
Anti-PSMA1 autoantibodies	Serum	AUC 0.904 for INS vs. controls; tracks proteinuria; validated in independent cohort	Diagnosis; autoimmune podocytopathy	[38]
Extrafollicular/atypical B cells	Blood	Expanded in active INS; ablated by rituximab; MZ-like resurgence marks post-RTX relapse	Mechanism; relapse monitoring	[39]
Plasma proteomic panel (VDB/ADIPOQ/MMP-2)	Plasma	AUC 0.78 for SRNS at presentation; validation cohort *n* = 37	Predicts steroid resistance	[40]

AUC = area under the curve; NPV = negative predictive value; IS = immunosuppression; MCD = minimal change disease; SSNS/SDNS/SRNS/FRNS = steroid-sensitive/-dependent/-resistant/frequently relapsing nephrotic syndrome; RTX = rituximab; MZ = marginal zone.

## Data Availability

No new data were created or analyzed in this study. All data discussed are available in the original published articles cited in this review.

## Supplementary Materials

The following supporting information can be downloaded at https://www.mdpi.com/article/10.3390/medsci14040448/s1, Table S1. Studies evaluating autoantibodies in pediatric idiopathic nephrotic syndrome; Table S2. Studies evaluating urinary biomarkers in pediatric idiopathic nephrotic syndrome; Table S3. Studies evaluating cytokines and chemokines in pediatric idiopathic nephrotic syndrome; Table S4. Studies evaluating immune cell signatures in pediatric idiopathic nephrotic syndrome; Table S5. Studies on further candidate biomarkers in pediatric idiopathic nephrotic syndrome; Table S6. Preferred Reporting Items for Systematic reviews and Meta-Analyses extension for Scoping Reviews (PRISMA-ScR) Checklist [47].
