# Supplementary material for: Beyond the Steroid Trial: A Scoping Review of Biomarkers for Pediatric Nephrotic Syndrome
Source: Med Sci (Basel). 2026 Jul 29;14(4):448. doi: 10.3390/medsci14040448 (PMC13515232; doi:10.3390/medsci14040448)
Supplement: Supplementary file 1 [file medsci-14-00448-s001.zip › medsci-4400343-supplementary.pdf]

Table S1. Studies evaluating autoantibodies in pediatric idiopathic nephrotic syndrome.

Author (Year)	Biomarker(s)	Study Characteristics	Key Findings	Diagnostic & Prognostic Value	Evidence Tier
**Anti-nephrin autoantibodies**					
Watts et al. (2022)	Anti-nephrin IgG	62 MCD patients; 30 healthy controls; 54 PLA2R+ membranous nephropathy controls; ELISA + immunoprecipitation; serum; renal biopsy	Anti-nephrin IgG detected in 29% of patients during active disease; absent in all controls. In 11 paired relapse/remission samples, antibodies present at relapse disappeared completely in remission. IgG deposits co-localized with nephrin on biopsy, confirming slit diaphragm targeting.	Seropositivity in active disease only; 100% seroconversion in remission in paired samples. No formal AUC reported.	A
Raglianti et al. (2024)	Slit diaphragm proteins (including nephrin)	128 children with MCD or FSGS; 48 adults for comparison; confocal IF and STED microscopy on biopsy; serum ELISA	IgG deposition along the slit diaphragm in 30% of MCD and 25% of FSGS cases; 77.8% co-localised with nephrin. Among SRNS: positivity in 27% of primary and 87.5% of secondary SRNS. Antibody-positive patients achieved remission with second-line immunosuppression at far higher rates than antibody-negative patients, and none progressed to kidney failure.	Remission: 92.3% in antibody-positive vs. 20% in antibody-negative SRNS. Kidney failure: 0% in positives vs. 51.7% in negatives. Positivity predicts both IS response and renal survival.	A
Hengel et al. (2024)	Anti-nephrin IgG	182 children with INS; 50 pediatric controls; immunoprecipitation (qualitative) + hybrid IP→ELISA (quantitative); serum	Anti-nephrin positivity in 52% overall, rising to 80% in children with nephrotic-range proteinuria and 90% in those actively nephrotic without immunosuppression. Only 1/50 controls was positive. Longitudinal tracking showed antibody levels moving in parallel with proteinuria.	Prevalence tracks disease activity: highest in active untreated disease, lowest in remission. Quantitative hybrid assay enables longitudinal monitoring; no formal sensitivity/specificity reported.	A
Hengel et al. (2025)	Anti-nephrin IgG	333 children with NS (101 SSNS, 67 SDNS, 103 non-genetic SRNS, 62 genetic SRNS); multicenter PodoNet cohort; immunoprecipitation (qualitative); serum	Positivity was highest in SSNS (68%) and declined progressively across phenotypes: SDNS 28%, non-genetic SRNS 14%, genetic SRNS 2%. Among active non-genetic SRNS patients, positivity was identified in 18% of those who subsequently responded to intensified immunosuppression and in none of those with multidrug-resistant disease.	Prevalence inversely correlates with steroid resistance. In non-genetic SRNS, positivity predicts response to intensified IS (18% responders vs. 0% multidrug-resistant). Absent in genetic SRNS, supporting use as a discriminator between immune-mediated and monogenic disease.	A
**Other podocyte autoantibodies**					
Ye et al. (2021)	Vinculin, SRSF9, proteasome α1,	341 children with idiopathic NS vs. 195 healthy controls + 438 disease	At least one autoantibody detected in 66% of patients; anti-vinculin was the most frequent	Titers correlated with 24-h proteinuria and declined with effective therapy. No	A

	aconitate hydratase 2, peptidyl-prolyl isomerase D, peroxiredoxin 1, F-actin capping protein β	controls (HSP, HSP nephritis, IgA nephropathy); cross-sectional; 2D electrophoresis, mass spectrometry, recombinant protein immunoassays; serum	(56%). Antibody-positive patients had higher proteinuria than antibody-negative patients. Titers declined in remission in steroid-sensitive cases but remained elevated in SRNS, suggesting that persistence of autoantibodies reflects ongoing immune activity.	formal ROC analysis; diagnostic thresholds not established.	
Liu et al. (2025)	Anti-PSMA1 (proteasome subunit α-type 1) autoantibody	54 newly-diagnosed INS children; normal controls and four disease-control groups (Kawasaki, HSP, HSP nephritis, IgA nephropathy; n = 50 each); independent validation cohort of 30 children; protein microarray + ELISA; serum; supporting mouse immunization, podocyte knockdown, and psma1-knockout zebrafish models; China	Serum anti-PSMA1 was significantly higher in INS than in every comparator group and correlated positively with proteinuria (r = 0.40), falling in remission. Punctate IgG co-localized with PSMA1 on patient biopsies, and animal/cell models reproduced podocyte injury and proteinuria, supporting a pathogenic role. Anti-PSMA1 extends the podocyte-autoantibody family beyond nephrin and vinculin.	Discriminated INS with AUC 0.904 (sensitivity 74.1%, specificity 98.0%) at a cut-off of 28.2 grayscale units; reproduced in an independent validation cohort (positivity 76.6%). Tracks disease activity; no clinically available assay.	A

Abbreviations: NS — nephrotic syndrome; INS — idiopathic NS; SSNS — steroid-sensitive NS; SDNS — steroid-dependent NS; SRNS — steroid-resistant NS; MCD — minimal change disease; FSGS — focal segmental glomerulosclerosis; PLA2R — phospholipase A2 receptor; HSP — Henoch–Schönlein purpura; IF — immunofluorescence; STED — stimulated emission depletion microscopy; IP — immunoprecipitation; IS — immunosuppression; ROC — receiver operating characteristic; AUC — area under the curve.

Table S2. Studies evaluating urinary biomarkers in pediatric idiopathic nephrotic syndrome.

Author (Year)	Biomarker(s)	Study Characteristics	Key Findings	Diagnostic & Prognostic Value	Evidence Tier
**Tubular injury markers**					
Choudhary et al. (2020)	uVDBP, uNGAL	56 children with idiopathic NS (28 SRNS, 28 SSNS) vs. 28 healthy controls; cross-sectional; urine ELISA (VDBP and NGAL, normalized to creatinine); India	Both uVDBP and uNGAL were markedly elevated in SRNS compared to SSNS and controls. uNGAL showed the highest likelihood ratio among the three markers tested, outperforming uVDBP in discriminating steroid resistance.	uVDBP: AUC 0.897, sens 82%, spec 89% (cutoff 303.8 ng/mL). uNGAL: AUC 0.901, sens 86%, spec 78% (cutoff 13.1 ng/mL) for SRNS vs. SSNS.	B
Pradeepchandran et al. (2022)	uNGAL	79 children with first-episode idiopathic NS; baseline uNGAL before steroid initiation; prospective with 8-week outcome follow-up; urine ELISA; India	Baseline uNGAL below 10 ng/mL strongly predicted steroid responsiveness by week 8. All 63 children with uNGAL ≤10 ng/mL achieved remission on standard prednisone. Seven of 16 children with uNGAL >10 ng/mL were ultimately SRNS.	uNGAL <10 ng/mL: 100% negative predictive value for steroid resistance in this cohort. Identifies likely steroid sensitivity before therapy initiation.	B
Lodeweyckx et al. (2021)	uEGF	98 children with proteinuric glomerulopathy (various biopsy findings) + 49 healthy, 20 Alport controls; serial sampling; urine EGF normalized to Cr; linear mixed models; Belgium	Children with nephrotic disease had significantly lower uEGF/Cr than healthy controls, indicating early tubular injury despite normal GFR. MCD showed a distinctive profile: higher relative uEGF than FSGS/other lesions without ACEi, and no uEGF response to ACE inhibitor introduction — in contrast to FSGS, where ACEi increased uEGF/Cr by 78%.	MCD vs. FSGS/other: 57% higher uEGF/Cr without ACEi (p=0.026). ACEi response absent in MCD, present in FSGS/other (+78%). Differential ACEi response may support histological stratification.	B
**Podocyte-derived markers**					
Hidayati et al. (2023)	Podocyte-derived microparticles (nephrin, podocin, podocalyxin)	33 children with active NS (initial or relapse) vs. 22 healthy controls; cross-sectional; early-morning urine; flow cytometry for podocyte protein-positive extracellular vesicles; Indonesia	Urinary podocyte protein microparticles were significantly elevated in NS patients compared to healthy controls. Microparticle levels correlated with proteinuria severity across all three podocyte proteins, with nephrin-MP showing the strongest correlation.	ROC AUC for NS vs. controls: nephrin-MP 0.895, podocin-MP 0.849, podocalyxin-MP 0.728. Nephrin-MP correlated with uPCR (r≈0.70, p<0.001).	B
**Immune activation markers**					
Hooman et al. (2022)	uCD80 (B7-1)	51 children with idiopathic NS (41% MCD, 24% FSGS, 35% not biopsied); subgroups: SSNS vs. SRNS and relapse vs. remission;	uCD80/Cr was markedly elevated in MCD relapse but not in FSGS relapse, pointing to CD80 upregulation as a mechanism specific to immune-mediated MCD pathology. The	MCD relapse vs. remission: 206 vs. 17 ng/g Cr (p=0.003). SSNS relapse vs. SRNS relapse: 402 vs. 124 ng/g	B

		urine ELISA (CD80/creatinine ratio); Iran	highest uCD80 occurred in steroid-sensitive patients in relapse; SSNS and SRNS were indistinguishable in remission, confirming uCD80 as a marker of active immune-driven proteinuria rather than a static disease signature.	(p=0.001). Distinguishes MCD from FSGS in active disease.	
Thy et al. (2021)	uSuPAR	92 children with NS (49 initial onset, 43 relapse); longitudinal follow-up; urine suPAR ELISA normalized to creatinine; Vietnam	Urinary suPAR/Cr was higher in active relapses than in new-onset NS or healthy controls. Among relapsing patients, SRNS had significantly greater suPAR than SSNS. The difference was not statistically significant in the new-onset subgroup, suggesting suPAR may be more informative in established relapsing disease.	In relapsing NS: SRNS vs. SSNS suPAR/Cr 882 vs. 533 pg/µmol (p=0.02). Cutoff 950 pg/µmol: sens 73.9%, spec 89.5%, AUC 0.70 for SRNS in relapse.	B
**Gene expression markers**					
Li et al. (2022)	Urinary sediment mRNAs (CREBBP, CYBA)	129 children with INS (94 SSNS, 35 SRNS); discovery: NanoString 770-gene panel (n=6); validation: RT-qPCR (n=129); urine sediment; China	CREBBP mRNA was significantly elevated in SRNS compared to SSNS while CYBA was significantly lower, suggesting divergent immune regulatory gene expression at the urinary level. Individually, both markers showed modest predictive power; their combination yielded a marginal improvement and awaits independent replication.	CREBBP: AUC 0.655, sens 83.3%, spec 49.4%. CYBA: AUC 0.669, sens 58.3%, spec 83.1%. Combined CREBBP+CYBA: AUC 0.666, sens 63.9%, spec 76.4%.	A

Abbreviations: NS — nephrotic syndrome; INS — idiopathic NS; SSNS — steroid-sensitive NS; SRNS — steroid-resistant NS; MCD — minimal change disease; FSGS — focal segmental glomerulosclerosis; VDBP — vitamin D-binding protein; NGAL — neutrophil gelatinase-associated lipocalin; uEGF — urinary epidermal growth factor; uCD80 — urinary CD80; suPAR — soluble urokinase plasminogen activator receptor; MP — microparticle; uPCR — urine protein-to-creatinine ratio; Cr — creatinine; ACEi — ACE inhibitor; AUC — area under the curve; sens — sensitivity; spec — specificity; NPV — negative predictive value; ROC — receiver operating characteristic; ELISA — enzyme-linked immunosorbent assay; RT-qPCR — reverse transcription quantitative PCR.

Table S3. Studies evaluating cytokines and chemokines in pediatric idiopathic nephrotic syndrome.

Author (Year)	Biomarker(s)	Study Characteristics	Key Findings	Diagnostic & Prognostic Value	Evidence Tier
**Interleukins and multiplex panels**					
Nickavar et al. (2020)	IL-1β, IL-2, IL-6, IL-8, IL-13, IL-18	48 INS children (21 SSNS, 27 SRNS) vs. 19 healthy controls; cross-sectional; serum ELISA; pre-IS sampling; Iran	IL-1β, IL-2, IL-6, and IL-8 were significantly elevated in INS compared to controls, while IL-13 and IL-18 were lower. Among phenotypes, SSNS showed higher IL-1β, IL-6, and IL-8 than SRNS, suggesting that a more pronounced pro-inflammatory cytokine milieu characterizes the steroid-sensitive phenotype at presentation.	IL-8: AUC 0.890 (sens 87.2%, spec 94.7%) for INS vs. controls. IL-1β: AUC 0.819 (sens 65%, spec 78.5%) for SSNS vs. SRNS. IL-8: AUC 0.681 for steroid response.	B
Agrawal et al. (2021)	27-cytokine multiplex panel (IL-7, IL-9, MCP-1 panel; IFN-γ, TNF-α, IL-5, IL-13)	40 new-onset NS children (26 SSNS, 14 SRNS); paired plasma pre-GC and ~7 weeks post-GC; 27-plex Luminex bead-based assay; USA	Thirteen cytokines differed between SSNS and SRNS before treatment. Glucocorticoid therapy reduced IFN-γ, TNF-α, IL-7, IL-13, and IL-5 in both groups, but phenotype-specific differences persisted post-treatment. A 3-cytokine panel (IL-7, IL-9, MCP-1) best discriminated steroid resistance at baseline.	IL-7/IL-9/MCP-1 panel: ROC 0.846, sens 0.643, spec 0.846 for SRNS prediction before treatment initiation.	A
**TNF-α**					
Gowtham et al. (2024)	TNF-α	112 children with idiopathic NS (1–14 years); serum at presentation and at remission or day 42 if SRNS; ELISA; India	Serum TNF-α fell significantly with treatment in first-episode NS and IFRNS, while SRNS showed a non-significant rise, suggesting that TNF-α dynamics — rather than its baseline level alone — may track steroid responsiveness. SDNS had the highest baseline TNF-α of all subgroups, attributed to cumulative podocyte injury. Higher baseline TNF-α was associated with a longer time to remission.	Each 5.47 pg/mL increment in baseline TNF-α corresponded to ~1 additional day to remission (p=0.037). SRNS subgroup limited to 4 patients; no formal AUC analysis. TNF-α dynamics informative but not yet a standalone predictor.	B
**High-dimensional cytokine profiling**					
Kopač et al. (2025)	CSF1, IL-4, FLT3LG, CCL19, MMP12, IL-17F, CCL13 (Olink Target-48 panel)	18 INS children (MCD predominant); plasma at three timepoints: onset/relapse, remission on corticosteroids, remission after steroid withdrawal; Olink Target-48 proximity extension assay; Slovenia	CSF1, IL-4, FLT3LG, CCL19, and MMP12 were significantly higher at onset/relapse than remission. CSF1 and IL-17F distinguished onset/relapse from post-taper remission. CCL19, MMP12, and CCL13 rose again after steroid discontinuation, suggesting incomplete suppression of	Paired longitudinal differences across three disease states; descriptive significance only. Small cohort (n=18) limits generalisability. No ROC analysis; markers are candidate indicators of steroid withdrawal risk rather than validated predictors.	C

inflammatory signaling after withdrawal — a potential early molecular signal of impending relapse.

Abbreviations: INS — idiopathic NS; NS — nephrotic syndrome; SSNS — steroid-sensitive NS; SRNS — steroid-resistant NS; SDNS — steroid-dependent NS; IFRNS — infrequently relapsing NS; GC — glucocorticoid; IS — immunosuppression; MCD — minimal change disease; ELISA — enzyme-linked immunosorbent assay; ROC — receiver operating characteristic; AUC — area under the curve; sens — sensitivity; spec — specificity; MCP-1 — monocyte chemoattractant protein-1; IFN-γ — interferon-gamma; TNF-α — tumour necrosis factor-alpha; IL — interleukin; CSF1 — colony-stimulating factor 1; FLT3LG — Fms-related tyrosine kinase 3 ligand; MMP12 — matrix metalloproteinase-12; CCL — CC chemokine ligand.

Table S4. Studies evaluating immune cell signatures in pediatric idiopathic nephrotic syndrome.

Author (Year)	Biomarker(s)	Study Characteristics	Key Findings	Diagnostic & Prognostic Value	Evidence Tier
**Multi-lineage flow cytometry**					
Ye et al. (2021)	T cells (CD4+, CD8+, Tregs), B cells (total, plasmablasts, transitional), NK cells, monocytes	85 children with idiopathic NS (59 SSNS, 26 SRNS) vs. 30 healthy controls; peripheral blood flow cytometry before and after corticosteroid treatment	Immune dysregulation was broad but phenotype-specific. SSNS was characterized by B cell expansion (elevated total B cells and plasmablasts) and depletion of activated HLA-DR+ Tregs. Both groups showed increased CD8+ T cells, with activated CD8+ T cells reduced selectively in SSNS. Non-classical monocytes were depleted across both phenotypes; NK cells showed a CD56hi/CD56low subset shift, more prominently in SSNS.	No formal diagnostic metrics reported. Findings are descriptive, identifying SSNS-specific patterns of B cell expansion and Treg depletion that may reflect disease-driving immune mechanisms.	B
Chen et al. (2021)	T cell subsets (CD3+, CD4+, CD8+), immunoglobulins (IgG, IgA, IgM), complement (C3, C4), uCD80, hs-CRP, IL-6	285 NS children (187 SSNS, 98 SDNS/FRNS) vs. 50 healthy controls; cross-sectional; peripheral blood flow cytometry + serum immunoassays + urine ELISA; pre-corticosteroid; China	Both NS groups showed suppressed CD3+, CD4+, and CD4/CD8 ratio. The CD4/CD8 ratio was further reduced in SDNS/FRNS compared to SSNS, indicating more pronounced T cell dysregulation in the relapsing/dependent phenotype. Urinary CD80 was higher in SSNS than SDNS/FRNS, while hs-CRP and IL-6 were higher in SDNS/FRNS. The IgG/IgM ratio — historically associated with steroid dependence — was also lower in SDNS/FRNS.	No AUC reported. SDNS/FRNS vs. SSNS: CD4/CD8 ratio 0.97 vs. 1.16 ($p<0.001$); IgG/IgM 2.87 vs. 3.82 ($p<0.001$); uCD80 5.21 vs. 7.72 ng/mL ($p<0.001$). Combined immune-marker pattern distinguishes relapsing/dependent from sensitive phenotype.	B
Deng et al. (2023)	T cell subsets (CD4+, CD8+), B cells (CD19+), NK cells (CD56+CD16+), CD8+T/CD19+B ratio	204 NS children (SSNS, SRNS, SDNS/FRNS) vs. 19 healthy controls; retrospective; peripheral blood lymphocyte subsets by flow cytometry at onset and remission; China	CD4/CD8 ratio was persistently reduced in SRNS at both onset and remission. CD8+ T cell expansion was most prominent in SDNS/FRNS at onset and normalized with treatment. B cell kinetics differed between phenotypes: CD19+ B cells increased in SRNS at onset but fell in remission, while the opposite pattern occurred in SDNS/FRNS. NK cell depletion was consistent across all NS groups. The CD8+T/CD19+B ratio was highest in SDNS/FRNS at onset.	CD19+ B cells for SRNS: AUC 0.718 (95% CI 0.624–0.812), cutoff 17.46%, sens 71.2%, spec 70.8%. CD8+T/CD19+B ratio for SDNS: AUC 0.805 (95% CI 0.726–0.884, $p<0.001$) at cutoff 2.22.	B

**High-dimensional B cell profiling**					
Fribourg et al. (2021)	38-marker CyTOF panel: class-switched B cell subsets (B-REG, switched memory resting/activated, CSM CD27−, antibody-secreting cells), T cell subsets, NK cells, monocytes	30 SDNS children receiving B cell-depleting therapy (rituximab or ofatumumab); longitudinal CyTOF at baseline, peak depletion, and relapse/stable timepoints; 17 relapsers vs. 13 stable; USA	Despite comparable B cell depletion between agents and between relapsers and stable patients, five class-switched B cell subsets were significantly more abundant in relapsers at both baseline and at the relapse timepoint. T cell subsets including Tregs and T follicular helpers did not differ. Class-switched B cell reconstitution was the dominant immunological signal associated with relapse risk after B cell depletion.	Five class-switched B subsets elevated in relapsers vs. stable patients ($p<0.05$ each). Composite switched B cell absolute numbers also higher at relapse timepoint ($p<0.05$). No AUC reported; findings establish biological rationale for monitoring switched B cells post-rituximab.	A
Riganati et al. (2024)	B cell subsets (transitional, plasmablasts, memory, unswitched memory), Tregs	44 new-onset INS children; sampled at diagnosis and 3 months post-prednisone; 12-month relapse follow-up; flow cytometry; 40 SSNS with relapse outcome data	At onset, patients had elevated total B cells, transitional B cells, plasmablasts, and memory B cell subsets versus controls, alongside reduced Tregs. Among SSNS patients, relapsers had higher transitional B cells, CD19+CD27+ memory B cells, and unswitched memory B cells at baseline. Unswitched memory B cells retained independent predictive value after age adjustment. Treg reduction was not normalized by prednisone treatment.	Unswitched memory B cells >1.5%: AUC 0.79 ($p=0.005$) for relapse within 12 months. OR 3.6 ($p<0.05$) after age adjustment. Best single baseline predictor of relapse in SSNS.	A
Al-Aubodah et al. (2023)	Extrafollicular B cells ($CD21^{low}$ $CXCR5^{-}$ $T\text{-}bet^{+}$ $CD11c^{+}$ atypical B cells; marginal-zone-like B cells; antibody-secreting cells)	Longitudinal cohort of 31 children with INS (active disease, glucocorticoid-induced remission, rituximab-maintained remission, post-rituximab relapse) and 24 healthy controls; single-cell RNA sequencing + multiparameter flow cytometry; Canada	Perturbation of the B cell transcriptional landscape was the dominant immune abnormality in active INS, driven by an extrafollicular response with expansion of atypical B cells and short-lived antibody-secreting cells. Glucocorticoids reduced antibody-secreting cells only, whereas rituximab ablated the full spectrum of INS-associated B cells; nascent resurgence of marginal-zone-like B cells marked post-rituximab relapse.	Mechanistically links rituximab-targeted B cell populations to podocyte-directed autoantibody generation and to relapse; identifies extrafollicular B cell subsets as candidate monitoring markers. No formal diagnostic metrics reported.	A
**Composite blood count indices**					
Jamee et al. (2022)	NLR, PLR	50 NS children across all phenotypes (36% SSNS, 30% SDNS, 22% FRNS, 12% SRNS); NLR and PLR from CBC before and after steroid therapy	Neither NLR nor PLR changed significantly with steroid treatment and neither was associated with steroid response category. These readily available CBC-derived ratios did not carry prognostic signal in this cohort, providing a useful negative result in	Negative result: NLR 1.9 vs. 2.2 ($p>0.05$); PLR 10.9 vs. 11.7 ($p>0.05$). No association with phenotype. NLR and PLR are not informative as biomarkers in pediatric NS at this time.	C

the context of their emerging use in other inflammatory conditions.

Abbreviations: NS — nephrotic syndrome; INS — idiopathic NS; SSNS — steroid-sensitive NS; SRNS — steroid-resistant NS; SDNS — steroid-dependent NS; FRNS — frequently relapsing NS; MCD — minimal change disease; CyTOF — cytometry by time-of-flight; Treg — regulatory T cell; NLR — neutrophil-to-lymphocyte ratio; PLR — platelet-to-lymphocyte ratio; CBC — complete blood count; uCD80 — urinary CD80; hs-CRP — high-sensitivity C-reactive protein; AUC — area under the curve; sens — sensitivity; spec — specificity; OR — odds ratio; CI — confidence interval.

Table S5. Studies on further candidate biomarkers in pediatric idiopathic nephrotic syndrome.

Author (Year)	Biomarker(s)	Study Characteristics	Key Findings	Limitations	Evidence Tier
**Stress-response and acute-phase proteins**					
Aksu et al. (2024)	HSP70 (serum, urine)	36 SSNS vs. 35 controls; prospective cohort; ELISA; serial sampling at days 0, 15, 30, 90; Turkey	Urinary HSP70 tracked disease activity and reflected glomerular injury burden, declining with steroid treatment and correlating with proteinuria severity. Serum HSP70 moved paradoxically in the opposite direction. Neither form distinguished frequent from infrequent relapsers, limiting prognostic utility within the SSNS phenotype.	Single centre; no SRNS comparator; relapse subgroups underpowered.	B
Pukajło-Marczyk et al. (2021)	Hemopexin (serum, urine)	51 INS children (onset n=20; relapses n=31) vs. 18 controls; case–control; paired relapse/remission; ELISA; Poland	Hemopexin behaved as a dynamic marker of disease activity, rising in relapse and falling — but not normalizing — in remission, suggesting persistent subclinical renal stress. Urinary hemopexin in remission was higher in children with prior relapses than in first-episode patients, pointing to a cumulative injury pattern. Treatment intensity influenced urinary but not serum levels.	Small control group; heterogeneous IS regimens; no diagnostic threshold reported.	B
**Circulating protein predictors of steroid resistance**					
Udagawa et al. (2021)	Serum IgM; selectivity index; total protein/albumin ratio	80 idiopathic NS children (13 SRNS); multicentre retrospective registry; routine serum assay; tertile analysis; Japan	Among readily available laboratory parameters, low serum IgM at diagnosis was the only independent predictor of steroid resistance, while the selectivity index and total protein-to-albumin ratio failed to discriminate SRNS from SSNS.	Retrospective; small SRNS subgroup (n=13); wide confidence intervals.	B
Wen et al. (2023)	ANGPTL3 (serum, urine)	200 NS (proteinuric n=144; other n=56) vs. 80 controls; cross-sectional; ELISA; serum and urine/Cr; China	Urinary ANGPTL3 normalized to creatinine proved the stronger diagnostic tool, substantially outperforming serum alone. Its behavior mirrored proteinuria and lipid burden, consistent with upregulation driven by hypoalbuminemia and nephrotic dyslipidemia, suggesting utility as a non-invasive marker of active disease.	Cross-sectional; heterogeneous NS cohort; no longitudinal or relapse data.	B

Agrawal et al. (2020)	Plasma proteomic panel (vitamin D-binding protein, adiponectin, MMP-2; hemopexin; apolipoprotein L1)	Paired pre- and post-treatment samples; discovery cohort of 15 paired samples; validation cohort of 37 patients (24 SSNS, 13 SRNS); label-free LC-MS/MS + immunoblot validation; plasma; Midwest Pediatric Nephrology Consortium, USA	A three-protein plasma panel (vitamin D-binding protein, adiponectin, MMP-2) predicted steroid resistance at disease presentation, while hemopexin and apolipoprotein L1 further distinguished steroid-sensitive from steroid-resistant disease. The signature independently converges on markers reported elsewhere in this review (urinary vitamin D-binding protein; hemopexin), lending cross-platform support.	Predictive panel differentiated SRNS from SSNS at presentation with AUC 0.78; companion proteomic study to the cytokine analysis of the same consortium.	A
**Metabolomics**					
Gooding et al. (2020)	Plasma metabolite panel (^{1}H-NMR)	45 children (30 SSNS, 15 SRNS); pre/post glucocorticoid (~7 weeks); ^{1}H-NMR plasma metabolomics; USA	Profiling before and after glucocorticoid therapy identified creatinine, glutamine, and malonate as candidate predictors of steroid resistance, pointing to perturbations in energy metabolism and redox homeostasis. The paired pre/post design was a methodological strength, but none of the candidates have been externally validated.	Small sample; post-treatment sampling confounds baseline profiling; no external validation.	C
Hu et al. (2023)	8-metabolite serum panel (D-mannitol, dulcitol, D-sorbitol, XMP, NADPH, NAD, bilirubin, α-KG)	27 SSNS vs. 56 controls (serum); validation: 41 SSNS vs. 43 controls; UHPLC-QTOF-MS + UPLC-MS/MS; treatment-naive only; China	Serum clearly discriminated SSNS from controls while urine profiling failed, establishing serum as the more informative matrix. Eight candidate biomarkers spanning glucose metabolism, purine pathways, and redox cofactors were causally linked to SSNS, with the most altered pathways implicating amino acid metabolism and the TCA cycle.	Single centre; SSNS only; small discovery cohort; AUC=1 suggests overfitting risk.	A
**Extracellular vesicles**					
Cricri et al. (2024)	EV surface marker panel (37 antigens; serum and urine)	105 INS children across disease states (onset/relapse/persistent proteinuria/remission) vs. 19 controls; cross-sectional; flow cytometry; Italy	EVs captured a multidimensional picture of disease activity, with urinary tetraspanins tracking proteinuria and a composite 8-marker signature distinguishing active from quiescent disease. Combining serum and urinary EVs improved staging precision, while specific urinary markers classified patients by steroid sensitivity — supporting EVs as a versatile, non-invasive biomarker platform rather than a single-analyte tool.	No prospective validation; flow cytometry not widely available clinically.	B

**Lipid profiles**					
Turolo et al. (2021)	Blood fatty acid profile (PUFA, ω-6, SFA, MUFA)	47 INS in stable remission vs. 47 matched controls; case–control; gas chromatography; whole blood; Italy	Even during clinical remission, children with INS showed a persistently pro-inflammatory lipid profile characterized by elevated omega-6 fatty acids and reduced saturated and monounsaturated fractions. This raises the hypothesis that residual lipid dysregulation may represent a subclinical vulnerability contributing to relapse susceptibility, independent of ongoing proteinuria.	Cross-sectional in remission only; no active disease comparisons; clinical significance unclear.	B
**microRNAs**					
Sinha et al. (2025)	Whole-blood miRNA panel (2,549 miRNAs; microarray)	12 SSNS (onset/relapse, off IS), 24 SRNS (at diagnosis, on prednisolone), 12 controls; prospective; Agilent microarray; whole-blood RNA; India	SSNS and SRNS were characterized by largely distinct miRNA expression profiles, suggesting differential gene regulation underpins divergent steroid response phenotypes. Target gene analysis pointed to pathways involving podocyte integrity, immune regulation, and NF-κB signaling as downstream effectors, though biological plausibility of individual miRNAs requires experimental confirmation.	Pilot study; small sample; SRNS patients on prednisolone at sampling; RT-PCR validation pending.	C

Abbreviations: NS — nephrotic syndrome; INS — idiopathic NS; SSNS — steroid-sensitive NS; SRNS — steroid-resistant NS; FSGS — focal segmental glomerulosclerosis; ANGPTL3 — angiopoietin-like protein 3; HSP70 — heat shock protein 70; Cr — creatinine; IS — immunosuppression; PUFA — polyunsaturated fatty acids; SFA — saturated fatty acids; MUFA — monounsaturated fatty acids; TCA — tricarboxylic acid; ¹H-NMR — proton nuclear magnetic resonance; UHPLC-QTOF-MS — ultra-high-performance liquid chromatography–quadrupole time-of-flight mass spectrometry; UPLC-MS/MS — ultra-performance liquid chromatography–tandem mass spectrometry; EV — extracellular vesicle; miRNA — microRNA; XMP — xanthosine monophosphate; NAD(PH) — nicotinamide adenine dinucleotide (phosphate); α-KG — alpha-ketoglutarate; AUC — area under the curve.

Table S6. Preferred Reporting Items for Systematic reviews and Meta-Analyses extension for Scoping Reviews (PRISMA-ScR) Checklist (Tricco et al., 2018)

SECTION	ITEM	PRISMA-ScR CHECKLIST ITEM	REPORTED ON PAGE #
**TITLE**			
Title	1	Identify the report as a scoping review.	1
**ABSTRACT**			
Structured summary	2	Provide a structured summary that includes (as applicable): background, objectives, eligibility criteria, sources of evidence, charting methods, results, and conclusions that relate to the review questions and objectives.	1
**INTRODUCTION**			

SECTION	ITEM	PRISMA-ScR CHECKLIST ITEM	REPORTED ON PAGE #
Rationale	3	Describe the rationale for the review in the context of what is already known. Explain why the review questions/objectives lend themselves to a scoping review approach.	5
Objectives	4	Provide an explicit statement of the questions and objectives being addressed with reference to their key elements (e.g., population or participants, concepts, and context) or other relevant key elements used to conceptualize the review questions and/or objectives.	5
**METHODS**			
Protocol and registration	5	Indicate whether a review protocol exists; state if and where it can be accessed (e.g., a Web address); and if available, provide registration information, including the registration number.	5
Eligibility criteria	6	Specify characteristics of the sources of evidence used as eligibility criteria (e.g., years considered, language, and publication status), and provide a rationale.	5
Information sources*	7	Describe all information sources in the search (e.g., databases with dates of coverage and contact with authors to identify additional sources), as well as the date the most recent search was executed.	5
Search	8	Present the full electronic search strategy for at least 1 database, including any limits used, such that it could be repeated.	5
Selection of sources of evidence†	9	State the process for selecting sources of evidence (i.e., screening and eligibility) included in the scoping review.	7
Data charting process‡	10	Describe the methods of charting data from the included sources of evidence (e.g., calibrated forms or forms that have been tested by the team before their use, and whether data charting was done independently or in duplicate) and any processes for obtaining and confirming data from investigators.	7
Data items	11	List and define all variables for which data were sought and any assumptions and simplifications made.	7
Critical appraisal of individual sources of evidence§	12	If done, provide a rationale for conducting a critical appraisal of included sources of evidence; describe the methods used and how this information was used in any data synthesis (if appropriate).	7
Synthesis of results	13	Describe the methods of handling and summarizing the data that were charted.	7
**RESULTS**			
Selection of sources of evidence	14	Give numbers of sources of evidence screened, assessed for eligibility, and included in the review, with reasons for exclusions at each stage, ideally using a flow diagram.	8

SECTION	ITEM	PRISMA-ScR CHECKLIST ITEM	REPORTED ON PAGE #
Characteristics of sources of evidence	15	For each source of evidence, present characteristics for which data were charted and provide the citations.	8
Critical appraisal within sources of evidence	16	If done, present data on critical appraisal of included sources of evidence (see item 12).	-
Results of individual sources of evidence	17	For each included source of evidence, present the relevant data that were charted that relate to the review questions and objectives.	8-18
Synthesis of results	18	Summarize and/or present the charting results as they relate to the review questions and objectives.	8-18
**DISCUSSION**			
Summary of evidence	19	Summarize the main results (including an overview of concepts, themes, and types of evidence available), link to the review questions and objectives, and consider the relevance to key groups.	18
Limitations	20	Discuss the limitations of the scoping review process.	20-21
Conclusions	21	Provide a general interpretation of the results with respect to the review questions and objectives, as well as potential implications and/or next steps.	22
**FUNDING**			
Funding	22	Describe sources of funding for the included sources of evidence, as well as sources of funding for the scoping review. Describe the role of the funders of the scoping review.	22
